# New Angiogenic Regulators Produced by TAMs: Perspective for Targeting Tumor Angiogenesis

**DOI:** 10.3390/cancers13133253

**Published:** 2021-06-29

**Authors:** Irina Larionova, Elena Kazakova, Tatiana Gerashchenko, Julia Kzhyshkowska

**Affiliations:** 1 Laboratory of Translational Cellular and Molecular Biomedicine, National Research Tomsk State University, 634050 Tomsk, Russia; kazakova.e.o@mail.ru; 2 Laboratory of Cancer Progression Biology, Cancer Research Institute, Tomsk National Research Medical Center, Russian Academy of Sciences, 634009 Tomsk, Russia; t_gerashenko@list.ru; 3 Institute of Transfusion Medicine and Immunology, Medical Faculty Mannheim, University of Heidelberg, 68167 Mannheim, Germany; 4 German Red Cross Blood Service Baden-Württemberg—Hessen, 68167 Mannheim, Germany

**Keywords:** tumor-associated macrophage, cancer, angiogenesis, OPN, SPARC, S100A, SEMA, VEGF, anti-angiogenic therapy, RTK inhibitor

## Abstract

**Simple Summary:**

Since the targeting of a single pro-angiogenic factor fails to improve oncological disease outcome, significant efforts have been made to identify new pro-angiogenic factors that could compensate for the deficiency of current therapy or act independently as single drugs. Our review aims to present the state-of-the art for well-known and recently described factors produced by macrophages that induce and regulate angiogenesis. A number of positive and negative regulators of angiogenesis in the tumor microenvironment are produced by tumor-associated macrophages (TAMs). Accumulating evidence has indicated that, apart from the well-known angiogenic factors, there are plenty of novel angiogenesis-regulating proteins that belong to different classes. We summarize the data regarding the direct or indirect mechanisms of the interaction of these factors with endothelial cells during angiogenesis. We highlight the recent findings that explain the limitations in the efficiency of current anti-angiogenic therapy approaches.

**Abstract:**

Angiogenesis is crucial to the supply of a growing tumor with nutrition and oxygen. Inhibition of angiogenesis is one of the main treatment strategies for colorectal, lung, breast, renal, and other solid cancers. However, currently applied drugs that target VEGF or receptor tyrosine kinases have limited efficiency, which raises a question concerning the mechanism of patient resistance to the already developed drugs. Tumor-associated macrophages (TAMs) were identified in the animal tumor models as a key inducer of the angiogenic switch. TAMs represent a potent source not only for VEGF, but also for a number of other pro-angiogenic factors. Our review provides information about the activity of secreted regulators of angiogenesis produced by TAMs. They include members of SEMA and S100A families, chitinase-like proteins, osteopontin, and SPARC. The COX-2, Tie2, and other factors that control the pro-angiogenic activity of TAMs are also discussed. We highlight how these recent findings explain the limitations in the efficiency of current anti-angiogenic therapy. Additionally, we describe genetic and posttranscriptional mechanisms that control the expression of factors regulating angiogenesis. Finally, we present prospects for the complex targeting of the pro-angiogenic activity of TAMs.

## 1. Introduction

Blood supply is crucial for the delivery of oxygen and nutrition components to a rapidly growing tumor mass [1,2]. Tumor progression frequently requires the transition from a quiescent to a proliferative vasculature named angiogenic switch [3]. One of the major drivers of tumor angiogenesis is hypoxia, a characteristic feature of rapidly growing tumor masses [1,2,3]. Tumor angiogenesis is defined as the formation of non-continuous endothelial structures characterized by high permeability for the metastatic cancer cells. Angiogenesis is a complex process that consists of distinct steps: (i) degradation of basement membrane; (ii) activation and migration of the endothelial cells (ECs); (iii) proliferation of endothelial cells; and (iv) formation of new blood vessels [4]. Tumor blood vessels are characterized by an aberrant morphology, including abundant branching, abnormal bulges and blind ends, discontinuous EC lining, and defective basement membrane and pericyte coverage [3]. Tumor angiogenesis is cancer type specific and affected by tumor grade and stage, by the cellular composite of tumor microenvironment, in particular the immune part, and by the balance in the pro- and anti-angiogenic factors [3]. 

Current widely used approach to target angiogenesis in cancer patients is based on the blocking of the main pro-angiogenic factor VEGF [5]. Despite the growing list of FDA-approved anti-VEGF drugs, the success of anti-angiogenic therapy is limited. Only short-term relief from tumor growth is detected, unfortunately followed by the development of resistance mechanisms, which remain under intensive investigation [1]. The limited efficacy of anti-angiogenic therapy based on the targeting of VEGF can be explained by the switching of the alternative pro-angiogenic activators leading to the development of tumor resistance during anti-VEGF therapy. Since the targeting of pro-angiogenic factor VEGF fails to improve oncological disease outcomes, significant efforts have been made to identify new pro-angiogenic factors that could compensate for the deficiency of anti-VEGF therapy or act independently as single drugs.

Tumor-associated macrophages (TAMs) are key cells in the tumor microenvironment (TME) that control angiogenesis [6,7,8]. The crucial role of TAMs in the angiogenic switch has been originally identified in a mouse model for breast cancer [9]. TAMs were found to secrete pro-angiogenic growth factors (first of all VEGF) and to facilitate the degradation of the perivascular extracellular matrix by a spectrum of released MMPs [10,11]. TAMs were identified both in murine models and patient samples as a potent source of different types of pro-angiogenic and extracellular matrix (ECM) degrading mediators, including VEGF, EGF, PDGF, TGF-α, and TGF-β, angiopoietin 1 and 2 (Ang-1 and -2), matrix metalloproteinases (e.g., MMP2, MMP9, and MMP12) and serine or cysteine proteinases, such as cathepsins and plasminogen activator (PA) [1,3,4,10]. Many “non-classical” growth factors, enzymes, ECM proteins, and other mediators produced by TAMs have been recently shown to regulate angiogenesis in animal models and in vitro, and in various types of human cancers. They include members of the S100 family, SEMA family, COX-2, SPP1 (osteopontin), SPARC (osteonectin), Tie-2, chitinase-like proteins (YKL-39, YKL-40), and others. 

Our review aims to present the state-of-the art for well-known and recently described factors produced by macrophages that induce and regulate angiogenesis. We summarize the data about the direct or indirect mechanisms of the interaction of these factors with endothelial cells during angiogenesis. We highlight recent findings that explain the limitations in the efficiency of current anti-angiogenic therapy approaches. Additionally, we describe genetic and posttranscriptional mechanisms that control the expression of factors involved in angiogenesis. We present prospects for the complex targeting of cancer angiogenesis, considering multiple factors and levels of regulation of this essential process for tumor progression.

## 2. Angiogenesis-Associated Factors Secreted by TAMs 

Angiogenesis is regulated by a great variety of factors secreted by both cancer cells and TAMs. The impact of this is most pronounced as a major problem for the development of immunotherapeutic and anti-angiogenic approaches in cancer. Here, we collected the data concerning new classes of pro- and anti-angiogenic growth factors, cytokines, matricellular proteins, and metabolic enzymes which are expressed by TAMs and could be new targets for anti-angiogenic therapy (Table 1). We classified angiogenesis-associated factors in four classes, namely the soluble mediators of cell–cell interactions, regulators of cell–matrix interactions, receptors, and intracellular enzymes.

### 2.1. Soluble Mediators of Cell-Cell Interactions

#### 2.1.1. S100A Family

**Figure 1:**
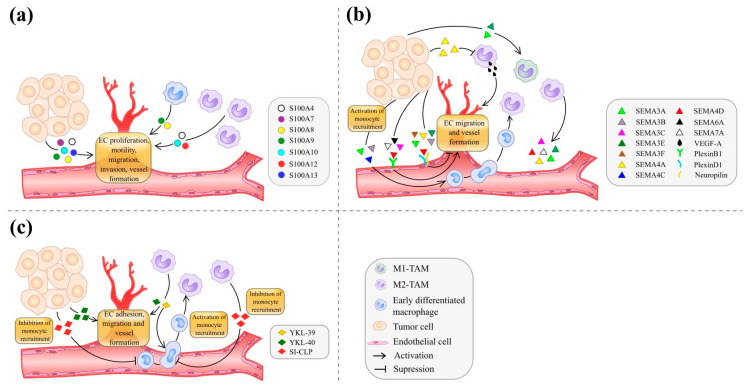
The role of TAM-produced soluble mediators of cell-cell interactions in tumor angiogenesis. They include the members of S100A family (**a**), of SEMA family (**b**) and of chitinase-like protein family (**c**).

Macrophages are able to produce several members of EF-hand calcium-binding cytosolic protein family, S100 [12] (Figure 1a). S100 proteins belong to damage-associated molecular pattern (DAMPs) proteins that regulate inflammatory responses and the recruitment of inflammatory cells to sites of tissue damage [13]. Major receptor classes responsible for pattern recognition are RAGE (receptor for advanced glycation end products) and TLRs (toll-like receptors) [14]. Several S100 proteins use the same receptor systems to transmit their signals. S100 proteins have a broad range of intracellular and extracellular functions, including the regulation of calcium balance, apoptosis, migration, proliferation, differentiation, energy metabolism, angiogenesis, tumor progression, and metastasis [12]. Clinical implications of S100 proteins in human cancers were found for the following members: S100A proteins, S100B, S100P, S100G [15]. As S100A proteins are the most investigated in tumor angiogenesis, we focused on them. 

The S100A protein family is comprised of 21 members of calcium-binding S100 proteins [16]. There is accumulating evidence concerning the angiogenic functions of macrophage-derived S100A proteins. We have collected data indicating that human macrophages produce the following S100A proteins: S100A4 (calvasculin, metastasin), S100A8 (calgranulin A; myeloid-related protein 8, MRP8), S100A9 (calgranulin B; MRP14), S100A10 (annexin A2 light chain), and S100A12 (calgranulin C, MRP6, or EN-RAGE).

Out of all S100A family members, S100A4 has the most pronounced and best investigated function in the regulation of cancer cell migration and metastasis. S100A4 expression was found in different human cell types, including monocytes, macrophages, fibroblasts, T lymphocytes, neutrophilic granulocytes, and endothelial cells (ECs). IHC analyses of tumor samples demonstrated the expression of S100A4 in lymphocytes, macrophages, endothelium, and smooth muscle cells [16]. No data regarding the role of S100A4-expressing macrophages in angiogenesis in different pathologies have been found.

S100A8 and S100A9 are predominantly expressed in monocytes, in early differentiated macrophages, neutrophils, and dendritic cells. They are also expressed in various other types of cells upon activation, such as fibroblasts, mature macrophages, vascular endothelial cells, keratinocytes, and cancer cells [12,13]. Depending on the presence of calcium, they can act as monomers, homodimers, heterodimers, or tetramers [13]. S100A8/A9 expression was identified in tumor-infiltrating CD68+ macrophages in human colorectal cancer [17,18]. S100A8/9 were up-regulated in M2-like THP-1 cells after stimulation by conditioned medium from myofibroblasts [17]. After stimulation, THP-1 cells are differentiated in vitro to the M2 phenotype, exhibiting increased expression of CD33, arginase-1, CD163, and CD206 [17]. S100A8 protein can activate the TLR4/NF-κB signaling pathway and aberrant expression of miR-155 in LPS-activated THP-1 macrophages [19]. Treatment of macrophages with LPS and S100A8 facilitated the migration of HCT116 and SW480 colorectal cancer cells in a transwell system in vitro [19]. For the S100A8 partnering protein S100A9, it was suggested that it is expressed by CCR2+ TAMs in hepatocellular carcinoma (HCC), where CCR2+ TAMs presented the same expression pattern as S100A9+ TAMs and were co-localized with CD31+ endothelial cells in areas of dense vascularisation [20]. However, direct evidence for S100A9 expression in CCR2+ TAMs has not been provided, and whether S100A8 is expressed in the same TAMs was not analyzed.

In primary human monocyte-derived macrophages, hyperglycemia, the hallmark of diabetes, elevates the expression of S100A9 and S100A12 during monocyte to macrophage differentiation by increasing the presence of activating histone marks on their promoters [21]. In human monocyte-like THP-1 cells, S100A12 expression is stimulated by IL-6 [22]. S100A12 expression was identified in neutrophils, monocytes, and macrophages in the early stages of differentiation (preferentially in inflammatory context) of endothelial cells, keratinocytes, and epithelial cells [12,21,23]. S100A10 is an essential molecule to convert plasminogen to plasmin, which is vital for fibrinolysis and angiogenesis, and is produced by macrophages, endothelial cells, and by the transformed cells [24]. In the Lewis lung carcinoma (LLC) mouse model, deficiency in S100A10 reduced macrophage recruitment to the tumor site [25]. S100A10-null TAMs were not able to stimulate angiogenesis and induce tumor growth compared to wild-type macrophages [25]. 

Macrophages are not the only source of S100 proteins elevated in various types of cancer. A number of studies demonstrated that cancer cells and ECs produce S100A proteins with pro-angiogenic activity. Thus, the Affymetrix microarray analysis of tumor and normal samples from patients with melanoma showed that S100A1 and S100A13 were expressed at a significantly higher levels in melanoma compared to normal skin tissue controls, while S100A2, S100A7, S100A8, S100A9, S100A10, and S100A11 were all highly expressed in primary melanoma samples compared to metastatic melanoma. Gene expression of these S100A proteins positively correlated with both lymphatic and distant site tumor metastases [26]. Analysis of a database with 1657 ovarian cancer patients showed that high expression of S100A2, S100A7A, S100A10, and S100A16 significantly correlated with worse overall survival (OS) in ovarian cancer patients, while the expression of S100A1, S100A3, S100A5, S100A6, and S100A13 were associated with better prognosis [27].

Increased protein expression of S100A4 correlates with a high incidence of metastasis and poor prognosis in cancer [28]. Oligomeric but not to dimeric form of S100A4 actively induces angiogenesis [29]. It was demonstrated that S100A4 stimulates the motility of endothelial cells, rather than their proliferation that can require other angiogenic factors to achieve a full angiogenic response [29]. The angiogenic action of S100A4 occurs in a cell-specific manner not affecting the motility of cancer cells and fibroblasts [29]. S100A4 acts through RAGE in human umbilical vein endothelial cells (HUVECs) to promote the migration of endothelial cells, and the pro-angiogenic action of S100A4 is synergistic with VEGF [30] (Table 2). Treatment with anti-S100A4 antibody 5C3 abolished the synergistic effect of the combination of VEGF and S100A4 on EC migration in vitro. In a mouse model of melanoma, silencing of S100A4 by siRNA reduced tumor growth and angiogenesis [30]. S100A4 stimulates angiogenesis by the calcium-dependent interaction of S100A4 and its effector protein MetAP2. Synthetic NBD peptide blocked the interaction between S100A4 and MetAP2 in endothelial cells and inhibited tumor angiogenesis and prostate cancer growth in tumor-bearing mice [31]. It was found that metastasis-associated protein 1 (MTA1) is involved in the regulation of S100A4 expression in endothelial cells by proteasomal degradation of S100A4 [32]. Silencing of both S100A4 and MTA1 in endothelial cells reduced tumor angiogenesis in vitro in MSS31 mouse endothelial cells and the formation of new blood vessels in vivo in xenografted mice inoculated with PANC-1 human pancreatic carcinoma cells [32]. 

The indirect action of S100A4 was also demonstrated. Overexpression of S100A4 in MDA-MB-231 breast cancer cell line up-regulated MMP13 expression resulted in an increased cancer cell migration and angiogenesis. siRNA-mediated silencing of S100A4 down-regulated MMP13 expression and suppressed cell migration and angiogenesis [33]. Down-regulation of S100A4 by siRNA in BCPAP and ML-1 thyroid cancer cells decreased cell invasion, metastasis, and angiogenesis, whereas S100A4 overexpression by cDNA transfection led to the opposite effect [34]. Silencing of S100A4 reduced VEGF and MMP-9 expression, as a possible mechanism of anti-angiogenic effect [34]. S100A4 may also be epigenetically regulated in colorectal cancer by tumor-suppressor miRs, miR-505c-5p, and miR-520c-3p, which promote S100A4-mediated migration and invasion of cancer cells [35].

Another member of S100 family, S100A7 (psoriasin), also induces the proliferation and migration of the endothelial cells and tube formation in vitro. The expression of S100A7 was induced by oxidative stress factors H2O2, hypoxia, and CoCl2, in HEKn cells (keratinocytes) [36]. Oxidative stress induced the expression of pro-angiogenic factors VEGF, HB-EGF, MMP-1, MMP-9, and IL-8, and reduced the expression of anti-angiogenic factor THBS-1 in S100A7-dependent manner [36,37]. 

S100A8 and S100A9 promoted the tube formation, proliferation, and migration of HUVEC cells in vitro via RAGE signaling and activation of mTORC2. The depletion of RAGE or Rictor, a target of rapamycin complex 2 subunit MAPKAP1, inhibited S100A8/9-induced angiogenesis [38,39]. 

Plasminogen receptor S100A10 was found to promote angiogenesis via GAS6/AXL pathway [40]. In human clear cell renal cell carcinoma (ccRCC) samples, increased S100A10 expression was associated with poor patient survival and positively correlated with AXL expression. In vitro, knockout of AXL in 786-O and M62 RCC cell lines reduced the expression of S100A10 and Annexin A2. HUVEC invasion and angiogenesis in the matrigel plug assay were activated by S100A10, while AXL inhibition reduced tumor progression and vessel density in ccRCC tumor xenografts and PDX models [40]. One more S100A member, S100A13, facilitated the release of FGF1 under heat shock conditions, modulating the proliferation of tumor endothelial cells synergistically effect with VEGF-A [41].

Thus, data obtained in animal tumor models and by the analysis of cancer patient material strongly indicate that S100A proteins regulate angiogenesis in cancer. The information regarding the expression of S100A proteins in human TAMs in situ and monocyte-derived macrophages in vitro is strictly limited and not available for all proteins. New findings may shed light on the mechanisms by which S100 proteins promote macrophage-mediated angiogenesis, and thus may provide a therapeutic target for tumor treatment.

**Table 1:** Macrophage-derived factors involved in the regulation of tumor angiogenesis.

Factors Regulating Angiogenesis	Pro- or Anti-Angiogenic Ability	Indirect Action Mediated by TAMs	Direct Action on ECs
S100A family			
S100A4	Pro-angiogenic	Not shown	Induces motility, migration of ECs, formation of new blood vessel in vitro [29,30,32].
S100A7	Pro-angiogenic	Not shown	Induces EC proliferation, migration and tube formation in vitro [36].
S100A8	Pro-angiogenic	Not shown	Induces EC proliferation and migration, and tube formation in vitro [38].
S100A9			
S100A10	Pro-angiogenic	Not shown	Activates ECs invasion and angiogenesis in vivo [40].
SEMA family			
SEMA3A	Anti-angiogenic	Suppresses angiogenesis via accumulation of M1-like macrophages in tumor in vivo [42].	Suppresses adhesion and migration of human ECs in vitro [43] and decreases average number of blood vessels in vivo [44].
	Pro-angiogenic	Induces angiogenesis by increasing TAM infiltration in hypoxic conditions in vivo [45].	ECs involved in neo-vessel sprouting secrete SEMA3A [43].
SEMA3F	Anti-angiogenic	Not shown	Inhibits EC adhesion and migration in vitro [43], decreases number of CD31+ cells in vivo [46].
SEMA3E	Anti-angiogenic	Induces pro-inflammatory polarization of macrophages [47].	Impairs tumor-induced blood vessel invasion into the angioreactors [48], decreases the number of filopodium-extending tip cells, disorganized vasculature [48] and decreases newly formed blood microvessels in vivo [49].
SEMA4A	Anti-angiogenic	Not shown	Suppresses EC migration and tube formation in vitro and decreases newly formed blood microvessels in vivo [49,50]. Suppressed VEGF-A–induced formation of new blood vessels in CAM assay [51].
	Pro-angiogenic	Stimulates expression and secretion of VEGF-A in macrophages [51]. SEMA4A-treated macrophages promote EC migration in vitro and increases vessel number and vessel branching in vivo [51]	Not shown
SEMA4D	Pro-angiogenic	TAMs are a main source of SEMA4D in breast cancer [52].	Induces EC migration and tube formation in vitro [53] and increases vessel formation in vivo [52,53].
SEMA6A	Pro-angiogenic	Not shown	Increases EC viability and growth in vitro, enhances network complexity and increases the number of vesel branching in vivo [54].
Chitinase-like proteins			
YKL-39	Pro-angiogenic	Increases monocyte recruitment. TAMs are a main source of YKL-39 in breast cancer [55].	Induces EC tube formation in vitro [55].
YKL-40	Pro-angiogenic	Not shown	Induces EC migration and spreading in vitro [56], and increases vascularisation in vivo [57,58].
Regulators of cell-matrix interactions			
OPN	Pro-angiogenic	Stimulates angiogenesis via TAM recruitment in vivo [59].	Promotes EC junctional destabilization, actin polymerization and EC motility in vitro and increases MVD in vivo [60].
SPARC	Anti-angiogenic	Inhibits angiogenesis via suppression of TAM recruitment in vivo [61].	Inhibits EC migration and vessel formation in vitro, decreases vessel number, and promotes disruption of the vascular basement membrane in vivo [62,63,64].
Other important angiogenesis regulators			
Tie2-receptor	Pro-angiogenic	TEMs induce tube formation and produce pro-angiogenic factors [65].	Promotes EC quiescence and vascular maturation in vitro [66].
COX-2	Pro-angiogenic	Enhances production of pro-angiogenic factors from TAMs [67].	Promotes EC migration, invasion and tube formation in vitro [59,68,69].

EC—endothelial cell; MVD—microvessel density; TAMs—tumor-associated macrophages, TEMs—Tie-expressing monocytes/macrophages.

#### 2.1.2. SEMA Family

Semaphorins (SEMA) are characterized by the presence of cysteine-rich SEMA-domain [70]. The SEMA family contains both membrane-bound and secreted proteins. Semaphorins were originally identified in the early 1990s as regulators of axon growth and guidance, essential for the development of the nervous system [71]. Besides neurogenesis, SEMA proteins are involved in various basic biological processes that include cytoskeleton remodeling, cell adhesion, migration, proliferation, and have specific effects on angiogenesis [72,73]. SEMA proteins are divided in eight classes [70,74]. SEMA from classes 1 and 2 and SEMA5C are found only in invertebrates. SEMA that belong to classes 3–7 are found only in vertebrates. Remarkably, SEMA were also found in viruses (class 8/SEMA V). Members of classes 1, 4, 5, and 6 are transmembrane proteins, members of classes 2, 3, and V classes are secreted proteins, and class 7 members are glycosylphosphatidylinositol (GPI)-linked [74].

SEMA can have both stimulating and inhibiting effect on angiogenesis that depends on the involvement of specific receptors. SEMA family members interact with plexin and neuropilin (Npn) receptors expressed on the surface of epithelial cancer cells, immune cells, ECs, and neural cells [75]. Angiostatic SEMA proteins include SEMA3A, SEMA3B, SEMA3F, interacting with NRP receptors, and SEMA3E, and SEMA4D, interacting with plexinD1 receptor. The angiostatic function is mediated by inhibiting integrin-mediated adhesion of endothelial cells to the extracellular matrix [70]. SEMA3C, SEMA4A, SEMA4D, SEMA6D, and SEMA7A possess pro-angiogenic properties via binding to plexin A and B receptors [70].

Several members of SEMA family are expressed by lymphoid and myeloid cells. Collected data indicate that macrophages can express SEMA3A, SEMA3C, SEMA3E, SEMA4A, SEMA4D, and SEMA7A [76,77,78,79]. Monocytes express SEMA3A, SEMA3C, SEMA3F, SEMA3E, and SEMA7A [47,76,78,80,81].

Class 3 semaphorins regulate macrophage-mediated inflammatory response and macrophage differentiation. Expression of SEMA3A and class 3 semaphorin receptors (plexin A1, A2, A3, and neuropilins NRP-1 and NRP-2) was found in human peripheral blood monocytes and was increased during differentiation into M2-like phenotype in response to M-CSF [76]. Human recombinant SEMA3A was shown to stimulate Fas-induced apoptosis in monocyte-derived macrophages [76]. In vitro recombinant SEMA3A enhanced LPS-induced IL-6 production in RAW264.7 macrophages [76]. SEMA3E-deficient mice treated with LPS demonstrated decreased inflammatory response mediated by macrophages. This effect was reversed in SEMA3A+/+ mice. In SEMA3E-deficient mice, the expression of pro-inflammatory cytokine TNF in bone marrow-derived macrophages (BMDMs) was decreased, and the number of CD11b+iNOS+ peritoneal macrophages as well as their phagocytic and Ag-processing ability were reduced comparing with WT mice [47].

Semaphorins regulate blood vessel morphogenesis, vascular development, and new vessel formation [74] (Figure 1b). The role of semaphorins in angiogenesis was demonstrated in both in vitro and in vivo models and is discussed below. 

Strong anti-angiogenic activity was shown for members of class 3 semaphorins that interact with neuropilin-1, -2 (NRP-1, -2), directly inhibiting EC functions [74]. In transwell system, recombinant human SEMA3A or SEMA3F inhibited both VEGF-A- and bFGF-enhanced adhesion and migration of human ECs to fibronectin and vitronectin, ligands of integrins [43] (Table 2). Anti-angiogenic effect of SEMA3A in VEGF-induced angiogenesis is mediated by inhibition of FAK and Scr signaling pathways in endothelial cells. Recombinant SEMA3A decreased average number of blood vessels in VEGF-stimulated Chick chorioallantoic membranes (CAM) assay [44]. Intraocular injections of human recombinant SEMA3E into P5 mice decreased the number of filopodium-extending tip cells, disorganized retinal vasculature with increased vessel diameter [48]. In a directed in vivo angiogenesis assay (DIVAA), VEGF- or bFGF-induced angiogenesis was reduced in response to human recombinant SEMA3E. Moreover, recombinant SEMA3E impaired head and neck cancer cell (HN12)-induced blood vessel invasion of ECs into the angioreactors in DIVAA assay [48]. Knockdown of plexin D1 by siRNA in HUVECs negatively regulated SEMA3E-induced cell-ECM adhesive via suppression of β1-integrin [48]. SEMA3E induced anti-angiogenic response in ECs via activation of plexin D1-Arf6 signaling axis [48]. 

SEMA4A was also found to inhibit angiogenesis. Recombinant soluble forms of SEMA4A (SEMA4A-Fc) and SEMA3E (SEMA3E-Fc) suppressed VEGF165-activated migration of HUVECs in transwell system and the formation of tubular structures in vitro [49]. SEMA4A-Fc or SEMA3E-Fc decreased newly formed blood microvessels within previously avascular areas in dose-dependent manner in a CAMs assay [49]. SEMA4A-deficient mice demonstrated increased blood vessel formation and elevated neovascularization in response to VEGF165 matrigel plug assays [49]. The direct anti-angiogenic effect of SEMA4A is mediated by its interaction with Plexin-D1 receptor on endothelial cells inhibiting VEGF-induced angiogenesis [49]. Knockdown of Plexin-D1 interfered to SEMA4A-mediated inhibition of HUVECs migration [49]. Conditioned medium from HSC-3 and SCC-25 cells (oral squamous cell carcinomas OSCC) transfected with SEMA4A CRISPR activation plasmid suppressed the tube formation of HUVEC cells and inhibited the expression of pro-angiogenic factors (VEGF and bFGF) in HUVECs [53].

Evidence concerning the pro-angiogenic activity of semaphorins has been also found. A chick chorioallantoic membranes (CAM) assay showed that ECs, involved in neo-angiogenesis, express SEMA3A. IHC analysis of VEGF-A-loaded matrigel plugs in adult mice showed that ECs involved in neo-vessel sprouting, secrete SEMA3A [43]. SEMA3A gene disruption resulted in the accumulation of vascular defects in the head and abnormal trunk blood vessels in homozygous E9.5 embryos of CD-1 mice [43]. 

SEMA4D activated angiogenesis via an interaction with endothelial receptor plexin-B1 [74]. The synergistic action of SEMA4D and VEGF promoted angiogenesis of A2780 and HUVEC endothelial cells via the SEMA4D/plexin-B1 pathway [50]. Vasculogenic mimicry analysis demonstrated that vessel formation was suppressed in SEMA4D-knockout HUVECs [50]. Co-transfection with SEMA4D and VEGF shRNA in HUVECs induced the migration of ECs in transwell migration assay [50]. 

A pro-angiogenic effect was also shown for SEMA6A. Suppression of SEMA6A in HUVECs with shRNA suppressed viability and VEGF-A-induced growth of ECs [54]. SEMA6A silencing reduced network complexity, decreased the extent of vessel branching and decreased VEGFR2 protein levels in SEMA6A-deficient gene-trap mouse line [54]. IHC analysis of matrigel plugs containing VEGF-A, FGF2, and heparin demonstrated a reduced number of CD31-expressing endothelial cells in SEMA6A-null mice compared with control mice [54]. In SEMA6A-deficient mice injected with VEGF-A and FGF2, decreased the number of CD31+ endothelial cells in matrigel plug assay was also observed [54]. 

Clinically, semaphorins correlate with the number of blood vessels and worse prognoses in cancer patients. Immunofluorescent staining of tumor tissues, obtained from 124 ovarian cancer patients, demonstrated the association of SEMA4D/plexin-B1 high expression with increased amounts of CD31+ cells [50]. Positive expression of SEMA4D in epithelial ovarian cancer was associated with poor OS and decreased progression-free survival (PFS) in comparison with samples of negative SEMA4D expression [50]. The role of SEMA3F in angiogenesis was shown in mouse model of colorectal cancer stably expressing recombinant SEMA3F. IHC analysis demonstrated that SEMA3F overexpression decreased tumor weight and number of CD31+ cells in comparing with control mice [46]. In SEMA6A-null mouse models of B16 melanoma and Lewis lung carcinoma (LLC), reduced vascularization and number of CD31+ cells were demonstrated in comparison with wild-type [54]. IHC analysis of OSCC specimens demonstrated that SEMA4A expression positively correlated with high microvessel density (MVD) and the poor prognosis in OSCC [53].

In the tumor microenvironment, semaphorins may skew monocytes into a pro-tumorigenic phenotype with increased production of pro-angiogenic factors supporting tumor growth. Human recombinant SEMA4A stimulated expression and secretion of pro-angiogenic factor VEGF-A in monocyte-derived macrophages through PlexinD1-mediated pathway in vitro [51]. Supernatants from SEMA4A-treated macrophages increased VEGFR-2 phosphorylation in ECs and promoted EC migration comparing to ECs incubated with conditioned medium (CM) of control cells [51]. While supernatants of macrophages treated with hrSEMA4A increased vessel number and vessel branching, purified hrSEMA4A suppressed the VEGF-A-induced formation of new blood vessels in a CAM assay [51]. In breast cancer mouse model, TAMs were demonstrated as a main source of SEMA4D that promoted ECs migration, vessel organization and tumor growth in vitro [52]. IHC analysis of 290 specimens from patients with gastric carcinoma, revealed significantly higher expression of SEMA4D and higher amount of CD68+ TAMs in comparing with normal tissues [82]. SEMA7A-silencing in a mouse model of mammary carcinomas induced the downregulation of pro-angiogenic proteins CXCL2/MIP-2, CXCL1, and MMP-9 in peritoneal elicited macrophages [83]. 

SEMA proteins can regulate the recruitment of macrophages to tumor site (Figure 1b). In breast cancer mouse model SEMA4C stimulated the recruitment of F4/80+ TAMs and up-regulated the expression of pro-angiogenic factors, angiogenin and CSF-1 [84]. In a mouse model of breast cancer, SEMA3A overexpression in 4T1 cancer cells decreased tumor volume to 61% and promoted the accumulation of M1-like (CD11b+Ly6G−lowMHCIIhigh) macrophages in the tumor compared to 4T1-control tumors [42]. SEMA3B induced IL-8-associated angiogenesis by increased TAM infiltration in a mouse model of melanoma [85]. The injection of IL-8 inhibitor (MAB208 antibody) in melanoma xenografts suppressed the recruitment of F4/80+ TAMs in a tumor that was associated with a decreased number of CD31+ cells [85]. 

Mechanisms by which TAMs regulate angiogenesis depend on the localization of TAMs in hypoxic or normoxic regions. In hypoxic areas of tumor hypoxia-induced SEMA3A attracts TAMs by activating VEGFR1 through the composition of neuropilin-1 (Nrp1) and plexinA1/plexinA4 [45]. TAM attraction to the hypoxic areas needs Nrp1/VEGFR1/PlexinA1/A4 whereas TAM retention requires PlexinA1/A4. Nrp1 deficiency inhibited TAM recruitment to hypoxic niches that abrogated tumor growth, metastasis, and angiogenesis and activated T-cell response in vitro and in vivo [45]. 

Thus, semaphorins regulate angiogenesis through the direct and indirect mechanisms, and major mediators of the indirect regulation are TAMs. Semaphorins affect TAM recruitment and profile of TAM-secreted inflammatory and pro-angiogenic factors that in turn control activation and migration of endothelial cells, and vessel organization supporting tumor growth. TAM can also express and secrete a spectrum of SEMA proteins, providing additional levels of control for tumor angiogenesis. 

#### 2.1.3. Chitinase-Like Proteins

Chitinase-like proteins (CLPs) are clear biomarkers for various types of macrophage activation and can be used as indicators for the primary stimuli responsible for the development of specific macrophage phenotypes [6,55,86,87,88,89] (Figure 1c). For example, IFNg stimulates production of YKL-40, while TGFbeta stimulates YKL-39 and suppresses YKL-40 [89,90,91]. YKL-39 was identified as a biomarker for specific stabilin-1+ TAM subpopulation in human breast cancer [55]. In a tumor, CLPs can be secreted by both cancer cells and other cells of the TME, such as macrophages, endothelial cells, and fibroblasts [88].

Several recent studies have demonstrated that chitinase-like protein YKL-40 regulates tumor angiogenesis. It was reported that YKL-40 promotes the migration and spreading of vascular smooth muscle cells in vitro [56]. Ectopically, YKL-40-overexpressing breast cancer and colon cancer were subcutaneously injected in mice, and vascularization was 1.8–2.0 fold increased when YKL-40 was produced compared to control tumors [57,58]. YKL-40-overexpressing tumors had four- and eight-fold larger tumor size compared to the control tumor [58]. In the same study HMVEC cells were stimulated with CM of glioblastoma U87 cells with YKL-40 siRNA. Knockdown of YKL-40 suppressed tumor angiogenesis by 44% and reduced tumor volume by approximately 30% compared to control counterparts [58]. Recombinant YKL-40 protein was also found to induce the angiogenesis of vascular endothelial cells in vitro [87]. Today, the combined targeting of pro-angiogenic factors VEGF-A and YKL-40 for the improvement of survival in patients with glioblastoma is under consideration [92].

It was recently identified in our laboratory that YKL-39 is a new pro-angiogenic factor [55]. YKL-39 has a high structural similarity to YKL-40 [89]. YKL-39 was found to combine the functions of monocytes as a chemotactic agent and stimulator of endothelial cell tube formation in vitro (Table 2). In human breast cancer, we found that stabilin-1+ TAMs but neither cancer cells, nor endothelial cells or fibroblasts express YKL-39. Elevated gene expression of YKL-39 was associated with a high frequency of distant metastasis and with no objective response to neoadjuvant chemotherapy (NAC) in breast cancer patients [55]. We also demonstrated that the absence of clinical response in patients with breast cancer after anthracycline-containing NAC correlated with M2+ macrophage phenotype (YKL−39−CCL18+ or YKL−39+CCL18−) [93]. Our previous work already suggested that CD68+ TAMs can support tumor angiogenesis, primarily before NAC, while stabilin-1+ TAMs rather contribute to the maintenance of lymphatic vessel density after NAC [94]. TAM-produced YKL-39 is a new link for the complex interactions between TAMs, NAC, and angiogenesis.

Stabilin-1 interacting chitinase-like protein (SI-CLP) is expressed in vitro by M2 macrophages stimulated with IL-4 and dexamethasone [95]. Stabilin-1, first identified as scavenger receptor, is abundantly expressed on TAMs [86,96,97,98]. In addition to the endocytic and phagocytic function, stabilin-1 mediates intracellular sorting of newly synthetized chitinase-like proteins, SI-CLP and YKL-39, from the biosynthetic compartment to the secretory pathway [55,95,99]. Ectopically expressed in cancer cells, SI-CLP suppresses the growth of murine breast adenocarcinoma by decreasing macrophage accumulation in the tumor mass [88]. Recombinant SI-CLP also inhibited murine BMDM and human monocyte migration induced by CCL2 in vitro [88]. However, a role of SI-CLP in angiogenesis remains to be identified.

### 2.2. Regulators of Cell-Matrix Interactions

#### 2.2.1. Osteopontin (SPP1)

Secreted phosphoprotein 1 (SPP1, OPN, osteopontin) is an integrin-binding matricellular protein that is involved in a number of physiological and pathological processes, including cell adhesion and migration, angiogenesis, host immune response, wound healing, neurodevelopment, and tumor metastasis [100,101]. 

The expression of OPN was found in activated macrophages, T cells, osteoclasts, hepatocytes, smooth muscle cells, endothelial cells, and epithelial cells [102]. OPN is up-regulated in macrophages in different pathological conditions, including cancer, pulmonary fibrosis, systemic sclerosis, and diabetic atherogenesis [103,104,105,106]. 

There are clear indications that OPN can be produced by TAMs in lung cancer, colorectal cancer, hepatocellular carcinoma, ampullary cancer, melanoma [59,103,107,108,109]. OPN was highly expressed in TAMs isolated from patients with an advanced stage lung adenocarcinoma [107]. Co-culture of PMA-treated THP-1 macrophages with A549 lung cancer cells induced OPN expression in macrophages and activated immunosuppressive M2 polarization via the up-regulation of PD-L1 [107]. When RAW264.7 cell were supplemented with CM of melanoma cells, the expression of OPN was significant increased [59]. IF double staining confirmed the co-localization of OPN with CD68 in human melanoma samples [59]. IF staining also indicated the co-localization of OPN and CD68 at the stromal area of tumor sections in patients with colorectal cancer [110,111].

The expression of CD44, which is a cell surface receptor for OPN, is down-regulated in non-small cell lung cancer (NSCLC) tissue when compared with paired normal lung tissue [109]. When CD44S, a CD44 isoform, was transfected to NSCLC cell line H322, the enhanced susceptibility of H322 cells to the macrophage cytotoxicity mediated by macrophage OPN was observed [109]. In vitro CD44-positive cancer cells HT-29 induced OPN expression in THP-1 cells in transwell co-culture system [110]. TAMs and EpCAM+CD44+ colorectal cancer cells were isolated from human tumor-derived colorectal cancer xenografts, and then cancer cells were inoculated into nude mice with or without TAMs. In tumor-derived mice inoculated by TAMs, OPN gene expression in TAMs was dramatically increased (almost eight-fold), compared with OPN levels in peritoneal macrophages. Immunohistological analysis of xenograft tumors revealed OPN expression was elevated in the tumor stroma and the tumor islands in the TAM-inoculated group [110]. 

**Figure 2:**
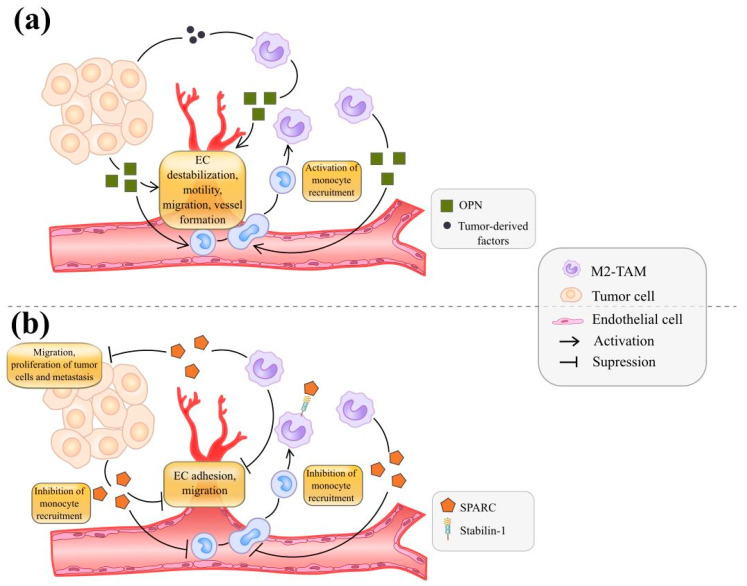
The role of TAM-produced regulators of cell-matrix interactions in tumor angiogenesis. They include osteopontin (OPN, SPP1) (**a**) and SPARC (**b**).

OPN also enhances the infiltration of macrophages into tumor tissue and their alternative activation [59,60,104,112,113,114] (Figure 2a). An OPN-knockout mouse model of chemically induced HCC demonstrated reduced numbers of F4/80+CD11b+TAMs, decreased expression of M2 macrophage markers ARG-1 and PD-L1, and increased levels of Th1 cytokines (IFN-γ, TNFα, CXCL10 and IL-12b) compared with tumors from control mice [113]. The amount of F4/80+CD11b+ TAMs and M2 macrophages (CD206+CD11b+) was decreased in OPN-deficient glioblastoma-bearing mice. Recruitment of TAMs into tumor tissues was suppressed in OPN−/− mouse model of melanoma [114]. In an OPN deficient LLC mouse model, the OPN of host origin induced macrophage recruitment into the cancer-affected pleural cavity [60].

OPN can directly and indirectly activate tumor angiogenesis in tumor mouse models in vivo and in vitro [18,60,115] (Figure 2a). In OPN-/- LLC mouse model, pleural vascular permeability was reduced in the absence of either host or tumor OPN, defining OPN as an enhancer of vascular leakage [60]. OPN destabilized endothelial cell junctional and activated angiogenesis in vitro [60] (Table 2). OPN increased MVD in MCF-7 xenografts in vivo, and anti-OPN antibody reduced the MVD in tumor [18]. In vitro OPN induced VEGF expression through interacting with integrin-αvβ3 and activating PI3K/AKT and ERK downstream pathways critical for migration and tube formation of HUVECs [18]. Exogenous and tumor-derived OPN increased the gene and protein expression of VEGF via Brk-dependent NIK/NF-κB–mediated ATF-4 activation in cancer cells. OPN-induced VEGF enhanced VEGFR-2 phosphorylation in ECs and EC motility in vitro as well as angiogenesis in vivo [115].

Macrophage-derived OPN can control angiogenesis. In murine macrophage-like cell line RAW264.7 the combination of IL-10+IL-18 induced pronounced overexpression of OPN (8-fold change for gene expression, 3,5-fold change for protein expression). (IL-10 + IL-18)-induced macrophages augmented the vascularisation ability of mouse endothelial b.End5 cells [116]. OPN induced angiogenesis by activating COX-2 expression through α9β1 integrin in macrophages in mouse model of melanoma [59]. In OPN−/− mice, F4/80+COX-2+ macrophages and MVD were decreased in tumor tissue as compared with OPN+/+ mice [59]. In vitro OPN-activated RAW264.7 cells stimulated EC migration and angiogenesis. In human melanoma tissue, OPN-expressed TAMs correlated with the amount of CD31+ blood vessels [59]. Similar results were obtained in CRC. The amount of F4/80+ TAMs in tumor tissue and the gene expression level of COX-2 were diminished in OPN-deficient mouse model of CRC [117].

According to collected data, OPN protein may directly regulate the function of endothelial cells (proliferation, motility, migration, and tube formation), promoting angiogenesis. The indirect action of OPN is mediated by its expression in macrophages and cancer cells resulting in increased angiogenesis that is shown in numerous animal models and in vitro systems. However, there is still not enough evidence about the mechanism of action of macrophage-derived OPN on angiogenesis.

#### 2.2.2. Anti-Angiogenic Protein SPARC (Osteonectin) 

SPARC (secreted protein acidic and rich in cysteine or osteonectin) is a soluble component of the extracellular matrix that controls cell–matrix interactions, cell migration, and cell proliferation [118]. In the tumor microenvironment, SPARC affects tumor growth, angiogenesis, and extracellular matrix deposition [119]. SPARC can be produced by both cancer cells and cells that form the TME [118] (Figure 2b). The clearest data about the role of SPARC in tumor angiogenesis are coming from investigations of gastric cancer, bladder cancer, pancreatic carcinoma, and neuro- and glioblastoma [61,62,119,120,121,122,123,124,125]. SPARC has both direct and indirect anti-angiogenic activity.

TAMs can both be a source of SPARC released as well as a system for the clearance of SPARC by receptor-mediate endocytosis (Figure 2b). SPARC is a ligand for stabilin-1, a scavenger receptor of alternatively activated macrophages [126]. It was proposed that M2 macrophages can coordinate extracellular matrix remodeling, angiogenesis, and tumor progression by regulating SPARC extracellular concentration via stabilin-1-mediated endocytosis of SPARC [126]. In gastric cancer, the main sources of SPARC are M2-like TAMs, defined as CD163+ and stabilin-1+ cells, and fibroblasts. Macrophage-derived SPARC reduced the ability of gastric cancer cells to migrate and proliferate in vitro and tumor growth in vivo [121]. IHC analysis of human gastric cancer demonstrated that SPARC protein expression was found predominantly in stroma surrounding gastric cancer cells, and SPARC expression was negatively correlated with VEGF expression and MVD [122]. In a stably transfected gastric cancer cell line, SPARC overexpression inhibited the expression of VEGF and MMP-7 and angiogenesis in vitro. However, this effect of SPARC was diminished by VEGF-neutralizing antibodies and MMP-7 knockdown in vitro [123].

During bladder carcinogenesis SPARC regulates invasion and metastatic spread. SPARC expression was found in stromal cells but not in cancer cells in Sparc+/+ mice with bladder cancer which had lung metastases. In Sparc–/– mice the incidence of metastasis was higher than in Sparc+/+ mice [119]. SPARC deletion promoted ROS generation and increased the production of pro-inflammatory and pro-angiogenic cytokines IL-6, CCL2, VEGF, and TNF-α as well as cytokines with pro-migratory ability, CCL3, CXCL2, CSF-1, and M-CSF, accompanied by greater tumor infiltration by mac1-positive TAMs [119].

The effectiveness of SPARC as an anti-angiogenic factor was demonstrated in combination with radiotherapy in neuroblastoma. SPARC reduced radiotherapy-induced angiogenesis by down-regulating VEGF-A via miR-410 as well as tumor size in subcutaneous mouse tumor models of neuroblastoma [124]. Using a mouse model of glioblastoma, massive infiltration of CD68+ macrophages with bubbly cytoplasm and increased expression of phagocytic cell markers (glycogen, glycoproteins, glycolipids, and mucins) were detected in Sparc-null tumors indicating that SPARC promotes phagocytosis of tumor cells [125]. In SPARC-overexpressed Daoy cells (medulloblastoma), suppressed vessel formation was observed compared to control Daoy cells [62]. SPARC overexpression led to the decreased expression of pro-angiogenic factors (VEGF, FGFR, EGF, and MMP-9) and MMP-9 activation reversed the anti-angiogenic effects of SPARC indicating essential role of MMP-9 in the SPARC-induced anti-angiogenic effect [62]. 

SPARC exerts its direct anti-angiogenic activity by the detachment of endothelial cells from substrata, inducing a rounded morphology of the EC in vitro through the tyrosine phosphorylation dependent pathway [63,64]. SPARC inhibits the mitogenic activity of VEGF by regulating the association of VEGF with its cell-surface receptors on ECs in vitro [127,128].

In a tumor model of pancreatic carcinoma, the absence of host SPARC promoted disruption of the vascular basement membrane, inhibition of pericyte recruitment, and reduction of MVD, resulting in enhanced vascular permeability and tumor supply by oxygen and nutrients for invasion and metastasis [61]. Monocyte recruitment and differentiation of macrophages to M2-like phenotype (CD163+/CD206+) were increased in Sparc−/− tumor-bearing mice [61]. Synthetic SPARC peptide inhibited bFGF-stimulated endothelial cell migration in vitro and angiogenesis in vivo. Decrease in the number of CD31+ endothelial cells and SMA+ pericytes was found in SPARC peptide-treated Matrigel plugs compared to the positive controls with bFGF alone [120]. In SPARC peptide-treated neuroblastoma xenografts, a reduction of the quantity of ECs as well as normalization of blood vessel architecture were detected compared to control [120].

Collected data indicate that SPARC can be expressed by both cancer cells and stromal cells, especially TAMs, and realize its anti-tumor activity by inhibiting angiogenesis and decreasing cancer cell invasion and metastatic spread.

### 2.3. Receptors

#### Tie2-Positive Macrophages and Monocytes

Perivascular TAMs that express the angiopoietin receptor Tie2 can promote tumor angiogenesis [129]. Tie2 (also known as Tek) was identified as an endothelium-specific tyrosine kinase receptor that regulates vascular maintenance (cell proliferation, migration, and stabilization), development of embryonic vascularization, and plays a significant role in pathological processes, such as tumor angiogenesis, atherosclerosis, and vascular leakage [130,131]. Tie2 is mainly expressed by endothelial cells in TME, but its expression on TAMs was also found [132,133,134]. Tie2-expressing macrophages (TEM) compiles the 20% of macrophages derived from CD14+ human blood monocytes [135]. Tie2 participates in angiogenesis through the regulation of inflammation and the recruitment of immune cells [136]. In more detail, Tie2 receptor binds to angiopoietin-1 or -2 (ANG-1 or -2), resulting in the formation of an ANG-Tie system that activates pro-inflammatory signaling pathways in macrophages and influences EC function [137]. Angiopoietins are a family of growth factors that are secreted by vascular smooth muscle cells, pericytes and ECs and are involved in vascular stabilization, cell survival, vessel homeostasis, and tissue repair [137]. ANG-1 and ANG-2 are context-dependent antagonists that induce EC activation [138]. ANG-Tie system functions as a regulator of EC state, where constitutive ANG1 promotes EC quiescence and vascular maturation, while acute ANG-2 antagonism induces EC sensitization to pro-angiogenic stimuli and consequent angiogenesis [66].

The formation of ANG-Tie complex resulted in JAK-STAT activation in human monocyte-derived macrophages stimulated by IFN-γ and IL-10. ANG cooperating with IFN-γ and IL-10 increased the expression of CXCL3, CXCL5, CXCL8, IL-6, and IL-12b in human macrophages in vitro [138]. Tie2-overexpressing mice in K/BxN serum transfer model of arthritis demonstrated increased IL-6 expression in BMDMs in response to Ang-2 stimulation [139]. THP-1 cells co-cultivated with HUVECs enhanced LPS-induced the suppression of proliferation and apoptosis of HUVECs via ANG-1 and NF-κB signaling pathways [140]. Tie2-positive and NRP1-positive macrophages promoted vascular network formation in immunolabeled embryonic hindbrain tissue [141]. 

It was shown that Tie2-positive monocytes are already pro-angiogenic in blood, however tumor-derived ANG-2 may amplify the pro-angiogenic and tumor-promoting activity of Tie-2-expressing TAMs [65]. Human TEMs isolated from fresh blood as well as TEM conditioned medium significantly induced tube formation in a HUVEC spheroid/sprouting assay when compared with Tie2 negative monocyte-conditioned medium [65]. TEMs expressed high levels of pro-angiogenic factors MMP-9 and VEGF, and M2 markers (COX-2, CD206, WNT5A). The proportion of F4/80+Tie2+ cells was greater than F4/80+Tie2− TAMs in ANG-2-overexpressing LLC mouse tumors [65]. Tie2-positive macrophages express higher levels of IL-10 than Tie2-negative macrophages in murine 4T1 tumors [142]. ANG-2-stimulated release of IL-10 by TEMs suppressed T cell proliferation and increased the proportion of CD4(+)CD25(high)FOXP3(+) Tregs. Genetic depletion of tumor TEMs significantly reduced the number of Tregs, indicating that TEMs is the immunosuppressive macrophage subpopulation in breast cancer [142].

Thus, Tie2-expressing macrophages represent a pro-angiogenic population of macrophages. Tie2 can be considered as a potential target for the anti-angiogenic therapy.

### 2.4. Intracellular Enzymes

#### COX-2 

Cyclooxygenase-2 (COX-2) is an enzyme that catalyzes the production of prostanoids and is crucial regulator of arachidonic acid metabolism [67,143]. In cancer, COX-2 contributes to cell invasion, proliferation, angiogenesis, and regulation of metastatic potential of cancer cells [144]. COX-2 expression was found in almost all cell types in the TME, including fibroblasts, endothelial cells, cancer cells, and cells of immune infiltrate [145,146], induced by diverse factors, such as mitogens, inflammatory mediators, and hormones [147]. TAMs abundantly express COX-2 [14,148].

Number of studies demonstrated that COX-2 is critical regulator of tumor angiogenesis. The impact of COX-2 on angiogenesis is indirect and is associated with the induction of pro-angiogenic factors. Overexpression of COX-2 in macrophages co-cultured with different breast cancer cell lines (MCF-7 and MDA-MB-231) promoted cancer cell proliferation and resistance to adriamycin-induced apoptosis [67]. In vitro COX-2 silencing in breast cancer cells co-cultured with TAMs inhibited cell migration, cell invasion, and angiogenesis via the suppression of pro-angiogenic factors VEGF and VEGFR [149]. The inhibition of COX-2 in CD11b+F4/80+ macrophages isolated from murine primary breast tumors, reduced expression levels of TGFβ, VEGF-A, and VEGF-C in TAMs [68].

M2 macrophages can regulate COX-2-dependent invasion of cancer cells and angiogenesis in human basal cell carcinoma (BCC). In vitro M2-polarized THP-1 macrophages and human monocyte-derived M2 macrophages co-cultured with BCC cells in a transwell system induced COX-2-dependent invasion of cancer cells and angiogenesis by induction of secretion of VEGF-A, bFGF and MMP-9 in BCC cells. When cancer cells were transfected with COX-2 siRNA, this effect was abrogated [150]. Osteopontin (OPN) stimulates the expression of COX-2 in macrophages, resulting in the induction of angiogenesis in melanoma [59]. Endothelial cell migration and angiogenesis in vitro were abolished, when macrophages were pretreated with COX-2 inhibitor, etoricoxib. Etoricoxib treatment remarkably suppressed MVD and the infiltration of macrophages into melanoma tissues, as compared with control mice. In human melanoma, infiltration of COX-2+ macrophage correlates with enhanced angiogenesis [59].

In HUVECs cultured with conditioned media from pancreatic ductal adenocarcinoma cells (PDAC), knockout of COX-2 suppressed their proliferation, migration, and tube formation [69]. Similar results were obtained in COX-2-knockout PDAC cells with decreased expression of VEGF [69]. 

Many studies of human cohorts indicated COX-2 as a strong contributor to angiogenesis. In patients with urothelial carcinoma IHC analysis revealed that high COX-2 expression correlated with high levels of the expression of macrophage marker CD68 and blood vessel marker CD34 [151]. Double staining demonstrated that TAM density and MVD were higher in COX-2 high-expression regions of tumor, and such areas were characterized by high HIF-1alpha expression. Thus, COX-2 can be involved in angiogenesis by regulation of TAM infiltration and hypoxia [151]. In IHC analysis of tissue samples of colorectal adenocarcinoma, a strong correlation between COX-2 and VEGF expression levels was found [152]. In the similar IHC studies in samples of urothelial carcinoma overexpression of COX-2 was associated with high expression level of HIF-1a and high amount of CD68+ TAMs that promoted tumor progression and angiogenesis through TAM infiltration and hypoxia in tumor sites [151]. IHC analysis of pancreatic adenocarcinoma and gastric cancer tissues also demonstrated the association between the expression of COX-2 and the MVD [153,154]. Using double immunostaining of colorectal cancer tissues, it was shown that CD68+ TAMs express COX-2 in region with high expression of mucin secreted by tumor cells [14]. The aggregation of TAMs was close to COX-2-postitive tumor nests in human basal cell carcinoma. A multivariate linear regression model revealed that both the number of TAMs and COX-2 expression in epithelial cells are significant predictors for invasion and angiogenesis [148].

Thus, COX-2 is considered a significant contributor to tumor angiogenesis mediated by macrophages. However, no previous studies have explained in detail the mechanism of COX-2-mediated pro-angiogenic effects in TAMs to elucidate the prospects for the further study of TAM pro-angiogenic functions in malignant diseases.

**Table 2:** The mode of direct action of recombinant proteins involved in angiogenesis.

Purified Factor	Source	Angiogenic Assay	Working Concentration	Angiogenic Effect	Reference
S100A4	Retrovirus-infected CSML0 cells	Cell motility assay	0.5 μM	Activates	[29]
S100A4	HCT-116 cell line	Migration assay	3 µM	Activates	[30]
S100A7	Abnova, catalog number is not specified	Proliferation, migration and tube formation assay	1 μg/mL	Activates	[36]
S100A8	Cyclex Co. Ltd., catalog number is not specified	Tube formation, proliferation and migration assay	10 μg/mL	Activates	[38]
S100A9	Cyclex Co. Ltd., catalog number is not specified	Tube formation, proliferation and migration assay	10 μg/mL	Activates	[38]
SEMA3A	R&D systems, catalog number is not specified	Adhesion and migration assay	200–700 ng/mL	Inhibits	[43]
SEMA3A	R&D systems, catalog number is not specified	CAM assay	50 μg/mL	Inhibits	[43]
SEMA3E	R&D systems, catalog number is not specified	DIVAA	100 ng/mL	Inhibits	[48]
SEMA3E	COS-7 cells	CAM assay	100 nmol/L	Inhibits	[49]
SEMA4A	COS-7 cells	CAM assay	100 nmol/L	Inhibits	[49]
YKL-39	Sino Biological Inc, catalog number is not specified	Tube formation assay	100 ng/mL	Activates	[55,87]
YKL-40	E. coli	Migration and tube formation assay	100 ng/mL	Activates	[58]
SPP1	Not indicated	Vascular permeability assays	10^−10^ M	Activates	[60]
SPARC peptides FSEN and FSEC	Chemically synthesized	Migration assay and matrigel plug assay	10 μM	Inhibits	[120]
CCL18	Not indicated	Tube formation assay	20 ng/mL	Activates	[155]

### 2.5. Other Pro-Angiogenic Factors Produced by Macrophages

There are many other pro-angiogenic factors which belong to different families of cytokines, enzymes, transcription factors, and non-coding RNAs. Below, we present examples of these factors.

#### 2.5.1. Hypoxia-Induced Factors

It is known that TAMs accumulate in hypoxic areas of tumor where they express high amounts of hypoxia-inducible factor-1 (HIF-1) contributing to tumor angiogenesis and invasion [7]. Using HIF-1α global knockout zebrafish model, it was found that macrophages are able to mobilize from the aorta-gonad-mesonephros in areas with vascular damage and to promote vascular repair, indicating HIF-1α as a significant regulator of interactions between macrophages and endothelial cells [156]. In a breast cancer spheroid mouse model infiltrated with HIF-1α−/− macrophages, the amount of CD206+ and stabilin-1+ macrophages was significantly increased compared to spheroids infiltrated by WT macrophages. WT but not HIF-1α−/− macrophages infiltrated into spheroids stimulated differentiation of embryonic stem cells to CD31+ cells, indicating the negative role of HIF-1a-depleted macrophages in promoting angiogenesis [157].

Hypoxic GLUT1-high TAMs displayed increased gene expression level of REDD1, mTOR inhibitor, compared to normoxic TAMs, that was demonstrated in murine models of lung cancer (LLC) and breast cancer (E0771 and PyMT) [158]. mTOR inhibition induced tumor vessel abnormalization and metastasis mediated by TAMs. In REDD1-knockout mice glucose uptake by TAMs was significantly higher than by ECs, while in WT mice glucose consumption was higher in ECs. The presence of REDD1-deficient TAMs stabilizes EC junctions and vessels, preventing metastasis [158]. An in vivo experiment with matrigel plugs containing supernatants of hypoxic LPS-stimulated macrophages demonstrated the IL-1-dependent increase in the number of blood vessels compare to supernatants of normoxic macrophages [159]. Neutralization of IL-1b completely prevented angiogenesis induced by hypoxic and normoxic macrophages, while neutralization of IL-1a mainly inhibited the angiogenic ability of hypoxic macrophages. A decrease in the number of blood vessels was found in Matrigel plugs containing supernatants of hypoxic IL-1a-deficient macrophages [159]. EC-produced lactate induces pro-angiogenic and pro-regenerative M2-like phenotype in murine model of ischemia. Loss of the glycolytic regulator PFKFB3 reduced lactate secretion by ECs, accompanying the diminished pro-angiogenic ability of macrophages due to a decrease of VEGF secretion and muscle regeneration [160].

#### 2.5.2. Chemokines and Cytokines

CCL18 produced by TAMs is an essential regulator of angiogenesis in breast cancer. CCL18-expressing TAMs mediated angiogenesis in vitro and in vivo by regulating migration and endothelial-mesenchymal transformation in endothelial cells via ERK and Akt/GSK-3β/Snail signaling [155]. Using neutralizing anti-CCL18 antibodies abolished TAM-dependent HUVEC migration. The combined silencing of CCL18 and VEGF synergistically diminished the pro-migratory effects of TAMs. In vitro, recombinant CCL18 induced the formation of endothelial tubular structures by HUVECs [155]. 

CXCL8 derived from human IL-4-stimulated THP-1 cells increased migration and invasion of bladder cancer cells and promoted formation of blood vessels in tube formation assay [161]. In a mouse model of thyroid cancer, the treatment with thyroid-stimulating hormone increased the production of VEGF-A and CXCL8 from tumor cells resulted in the recruitment of F4/80+ macrophages and in supporting angiogenesis and tumor growth [162].

The CXCL9, -10, -11/CXCR3 axis in macrophages regulates antitumor immune response [163]. M1 macrophages suppressed angiogenesis and growth of CRC and pancreatic ductal adenocarcinoma (PDAC) cells through the enhanced production of CXCL9, CXCL10 and CXCL11 in “tumor-on-chip” system in vitro [164]. In a mouse model of LLC, deletion of cancer-associated miR-21 in TAMs led to the increased tumor cell death and inhibition of neovascularization mediated by TAM-produced CXCL10 and IL-12 [165].

CXCL16 expression in thyroid cancer cells is associated with high expression of M2 polarization markers and pro-angiogenic markers, and positively correlates with poor prognosis in human papillary thyroid cancer [166]. In vitro, CXCL16 induced THP-1 migration. The application of anti-CXCL16 neutralizing antibody decreased the number of TAMs and ECs in a macrophage-laden xenograft tumor model. The authors suggested that depletion of CXCL16 in cancer cells and other cells of the TME can be an effective therapeutic strategy for advanced thyroid cancer [166].

#### 2.5.3. Non-Coding RNA 

Long non-coding RNAs (lncRNAs) regulate infiltration and polarization of macrophages and inflammation by targeting various pathways responsible for the pro- and/or anti-inflammatory responses [167]. lncRNAs can modulate the ability of macrophages to regulate angiogenesis.

Long non-coding RNA MALAT-1 (metastasis associated lung adenocarcinoma transcript 1) was recently found to be involved in the progression of thyroid cancer [168]. High levels of expression of MALAT-1 and FGF2 were detected in M2-polarized THP-1 cells and TAMs (THP-1 cells stimulated by CM from thyroid cancer FTC133 cells) in vitro. MALAT-1-dependent FGF2 secretion by TAMs inhibited the secretion of pro-inflammatory cytokines, promoted proliferation, migration, and invasion of thyroid cancer cells and increased angiogenesis [168]. MALAT1 and VEGF-A expression is up-regulated in hepatocellular carcinoma (HCC). Knockdown of MALAT1 in HCC cells significantly decreased VEGF-A expression in HCC cells, diminished the polarization of macrophages toward the M2 subset and inhibited angiogenesis of HUVECs in vitro [169].

lncRNA CRNDE overexpression resulted in the increased expression of CD163, VEGF, IL-10, TGF-β1, CCL22, and CCL24 in M2-polarized THP-1 cells [170]. M2-polarized THP-1 cells with CRNDE overexpression induced HUVEC cell viability, migration, tube formation in vitro in comparison with THP-1 cell without any stimulation. Blood vessel formation was also increased by CRNDE-overexpressed THP-1 cells in CAM membrane assay [170]. LncRNA MM2P was up-regulated in M2-polarized RAW264.7 cells and BMDMs, but was down-regulated in M1-polarized macrophages. Conditioned medium from both IL-13- and IL-4-treated RAW264.7 cells and BMDMs increased the extent of luminal formation by HUVEC cells in vitro [171].

## 3. Genetic and Posttranscriptional Alterations in Angiogenic Factors in Cancer

Genetic features including chromosome abnormalities, polymorphisms, point mutations or alternative splicing variants can affect activity of pro-angiogenic factors. 

Two isoforms of VEGF-A can be formed due to the alternative splicing: the pro-angiogenic VEGF165 isoform and the anti-angiogenic VEGF165b isoform. The balance between them defines the efficiency of angiogenesis and affects the results of anti-VEGF therapy in cancer [165,169]. Anti-angiogenic VEGF isoform is predominantly expressed in the majority of healthy tissues. In tumors, it is downregulated, resulting in the increased MVD that correlates with tumor progression [165,167]. Diversity of germline variants was described in regulatory region of VEGFA gene [172,173,174]. Among them, −2578A > C (rs699947) polymorphism and A2578-T936-T460 haplotype are associated with increased risk of cancer development and can be used as predictive markers for anti-VEGF-targeted therapy [175]. A whole-genome aCGH study of osteosarcoma samples demonstrated the association of copy number aberrations in 13 genes from VEGF pathway, including VEGFA amplification. These alterations resulted in the increased VEGF-A protein expression in tumor tissue and in the increased MVD [176].

Members of S100 family contribute to tumor angiogenesis [173]. Point mutations, translocation, and other chromosomal alterations in S100 genes are rare events in human cancers, and the main regulatory mechanism of tumor growth by S100 proteins is associated with changes in their expression levels. Association of S100 genes’ abnormalities with tumor progression was also demonstrated [177,178,179,180]. The deletion of chromosomal region 1q21, harboring S100A1, S100A2, and S100A14 genes, was found in 70% of patients with oral cancer that correlated with decreased expression of corresponding proteins [174]. Two polymorph variants in S100A14 gene, 1545A > T (rs11548102) and 461G > A (rs11548103), were described [174]. The latter one is associated with increased risk of esophageal cancer [181]. Several intron polymorphisms (144 + 109C > G, 297 + 17T > C, 297 + 75A > G), exon (185G > A), and a point mutation (67C > T) were described in NSCLC, but their functions in carcinogenesis are not yet investigated [177]. In gastric cancer, a single nucleotide polymorphism of S100A4, c.29A > T (rs1803245), significantly correlates with the reduction in cell migration ability of human gastric carcinoma cell lines [178]. However, the direct impact of S100 genetic variants on the efficiency of angiogenesis in various types of cancer still remains to be identified. 

Genetic variations and alternative splicing of osteopontin (SPP1 gene), a regulatory matricellular protein, are frequent events contributing to the development and progression in various cancers [179]. Today, eight different isoforms of OPN protein are described, three of them are best-characterized and associated with worse tumor prognosis. Alternative isoforms of OPN, OPNa, OPNb, and OPNc, are presented in tumor tissue of breast cancer, non-small cell lung cancer, hepatocellular carcinoma, pancreatic cancer, esophageal cancer, and gastric cancer [179]. OPNc level positively correlates with the migration and invasion of tumor cells in glioma [180], ovarian cancer [182], gastric cancer [183], and lung cancer [184]. SPP1 gene, encoding osteopontin, contains 10 short deletions and insertions and approximately 300 SNP variants [179]. Among them, most frequent events in cancer are promoter polymorphisms, −156G > GG (rs17524488), −443C > T (rs11730582), −66T > G (rs28357094), associated with poor prognosis in breast cancer [185], gastric cancer [186], and glioma [179]. Overall, the landscape of SPP1 SNPs is very complex and, together with alternative splicing variants, can be substantially involved in cancer progression. The role of SPP1 genetic variants in blood vessel formation remains to be identified.

Semaphorins are essential regulators of tumor growth and metastasis [187]. There is limited information about the genetic alteration of SEMA genes. In lung cancer, a loss of two chromosome regions containing semaphorins 3p21.3 (SEMA3B and SEMA3F) and 5q21–22 (SEMA6A) is associated with the inhibition of proliferation and invasion of tumor cells and angiogenesis [188,189,190]. Several missense polymorphic variants, resulted in amino acid replacement in protein structure, were also found for SEMA3B—Thr415Ile (rs2071203), Arg348Cys, and Asp397His [189]. Allele variants in SEMA3B (rs2071203) and SEMA3F (rs2072054) genes correlate with poor prognosis of prostate cancer in the Hispanic population [191]. It is highly interesting to understand how SEMA allele variants affect angiogenesis in general and in solid tumors. 

Osteonectin (SPARC gene) is basic matricellular protein that plays a regulatory role in the tumor microenvironment, frequently together with OPN. SPP1 rs4754 genotype interacts with SPARC SNPs, rs1054204, rs3210714, and rs3549, via epistatic mechanisms that increases the risk of the development of gastric cancer [192]. Without the genetic influence of SPP1, polymorph variants of SPARC (rs1054204, rs3210714, and rs3549) had no effect on gastric cancer risk [192]. An individual SPARC polymorphism c.*1103G > A (rs1059829), allele G, significantly correlated with tumor recurrence in gastric cancer [193]. SPARC polymorphisms c.*1200G > A (rs3210714) and c.331-59 A > C (rs7719521), genotypes AG + GG and AC + CC, respectively, were significantly associated with higher VEGF protein expression in breast cancer tissue compare to AA genotypes, which may explain the correlation with higher breast cancer risk and worse prognosis [194]. The genetic variants of SPARC gene, GA and GG rs3210714, were also associated with worse OS in pancreatic cancer patients [195]. The correlation between the SPARC genetic variants and efficiency of tumor angiogenesis was not addressed in these studies.

Tie2, a tyrosine kinase receptor in angiogenic endothelial cells and M2 macrophages, can bind angiopoietin and promote angiogenesis [196,197]. Polymorphic variants of TEK gene c.1521A > G (rs639225) GA/AA were associated with unfavorable OS of head and neck squamous cell carcinoma either among all patients, or in patients treated with the combination treatment (radiotherapy and cisplatin-based chemotherapy) [198]. The genotypes ANGPT2 (rs3739391) GA/AA, ANGPT2 (rs3020221) CC, TEK (rs639225) GA/AA, and VEGF (rs2010963) CC was related to decreased OS in patients with head and neck squamous cell carcinoma treated with the combination therapy [198]. Two TEK somatic mutations, c.2690A > G (rs80338909) and c.2743C > T (rs539652641) were found in hemangiomas [199]. The connection between TEK alterations, angiogenesis, and macrophage polarization still remains to be investigated. 

COX-2 is directly involved in mechanisms of carcinogenesis such as invasiveness, adhesion, and angiogenesis [66]. Due to the high heterogeneity in sequence variations of PTGS2 gene, coding COX-2, diverse pathological conditions are associated with different PTGS2 variants. The most frequent polymorphisms in PTGS2, which are associated with the development of gastric cancer, colorectal cancer, bladder cancer and breast cancer, are located in the promoter regions −1195G > A (rs689466) and −765 G > C (rs20417) [200,201,202,203,204,205]. The grade of T8473C allele SNP + 8473T >C (rs5275) is determinative for the PTGS2 expression level while C allele contributes to PTGS2 overexpression. SNP + 8473T > C was identified in a number of cancers including pancreatic cancer [206], esophageal cancer [207], colorectal adenoma [208], where COX-2 overexpression is a tumor-promoting factor [209]. Moreover, the polymorphism of PTGS2 serves as a potential marker for the efficacy of therapy with a nonsteroidal anti-inflammatory drug and selective COX-2 inhibitor [210]. Authors do not provide the information for role of PTGS2 sequence variations in angiogenesis.

Overall, there are an extensive number of specific genetic alterations which may affect the expression and clinical significance of angiogenic factors, derived from TAMs. Individual variations in gene sequencing, alternative splicing and chromosome abnormalities may partially explain the variants of the formation of tumor angiogenesis in cancer patients. Despite the number of genetic alterations and variants that were already identified for the pro-angiogenic factors, the impact of these variants on the pro-angiogenic activity of specific factors, as well as the correlation of these genetic variants with tumor vascularization remain almost unexplored area. Nevertheless, combinations of genetic and posttranscriptional alterations within one or several genes may give an impact on the final phenotype of tumor and should be taken into account during the development of personalized anti-angiogenic therapeutic schemes.

## 4. Perspectives for Anti-Angiogenic Therapy: Single and Combination Therapeutic Approaches

In clinical practice, anti-angiogenic drugs are administered to patients in both first- and second-line therapy [211,212,213,214]. Bevacizumab (Avastin), a VEGF-A targeting monoclonal antibody, was the first angiogenesis inhibitor approved by FDA in 2004 for the treatment of metastatic colorectal cancer (mCRC) in combination with chemotherapy [211,212]. Today indications for Bevacizumab application include metastatic breast cancer (mBC), non-small-cell lung cancer (NSCLC), glioblastoma, renal cell carcinoma (RCC), ovarian cancer, and cervical cancer [212]. Other FDA-approved angiogenesis blocking agents include inhibitors of receptor tyrosine kinases (RTK) regorafenib (approved for the treatment of refractory mCRC), ramucirumab (for cancers of gastro-intestinal tract, NSCLC, mCRC), sorafenib (for RCC, hepatocellular carcinoma (HCC), thyroid cancer), sunitinib (for RCC, pancreatic neuroendocrine tumours), pazopanib (for RCC, soft tissue sarcoma), axitinib (for RCC), vandetanib (for thyroid cancer), lenvatinib (for thyroid cancer), nintedanib (for NSCLC), and others [213].

Despite the growing list of FDA-approved drugs, the success of anti-angiogenic therapy has been limited, providing only short-term relief from tumor growth before resistance develops [1]. In accordance with the ability to respond to anti-angiogenic therapy, tumors are classified into sensitive tumors (RCC, ovarian and cervical cancer, HCC, thyroid cancer, neuroendocrine cancer), partly sensitive tumors (cancers of gastro-intestinal tract, BC, NSCLC, glioma), and resistant tumors (pancreatic cancer, prostate cancer) [213,214]. 

The limited efficacy of VEGF-targeted therapy can be explained by the switching on the alternative pro-angiogenic activators leading to the development of tumor resistance. The most common proposed mechanism of the resistance of angiogenesis-targeted therapy is related to the increased level of hypoxia in tumors. Hypoxia elevates level of HIF1a, which activates alternative pro-angiogenic growth factors [214]. The authors of these review articles suggested that infiltration of tumors with bone-marrow-derived cell populations can also stimulate angiogenesis in a VEGF-independent manner [213,214]. Besides, it was reported that tumor vasculature has six different blood vessel subtypes, of which four are VEGF-independent [215]. Such vascular heterogeneity is the basis of the search for alternative pro-angiogenic molecules that can serve as new targets.

Currently developed inhibitors of angiogenesis can be classified into two major groups: direct inhibitors that target endothelial cells in the growing vasculature, and indirect inhibitors affecting the expression and activity of the inducers of angiogenesis [216,217]. The indirect inhibitors include agents for targeted therapy against RTLs, pro-angiogenic factors, oncogenes, conventional chemotherapeutic agents, and drugs targeting immune cells in TME [216]. We summarized the data about the main mechanisms of action of the most investigated anti-angiogenic agents and their associations with TAM activity (Table 3). We highlighted the problem of single anti-angiogenic therapy and possibility of the increased effectiveness by combination several anti-angiogenic and TAM-targeting approaches.

### 4.1. The Effect of Direct Inhibitors on TAM Amount and Repolarization

The mechanism of action of the most known direct inhibitors (endostatin, canstatin, and tumstatin) is associated with the suppression of proliferation and migration of endothelial cells via the influence on the signal pathways in ECs [216].

Endostatin, is a recombinant human protein and the 20-kD C-terminal fragment of collagen XVIII [218,219]. Endostatin exhibits its anti-angiogenic action via suppression of endothelial cell proliferation and induction the apoptosis in ECs through modulation of ATPase activity [218]. In an A549-GFP xenograft tumor model, endostatin treatment suppressed tumor growth by increasing the number of apoptotic tumor cells and by inhibiting tumor angiogenesis, defined as a decreased number of CD31+ cells, compared with WT mice [219]. 

The effect of endostatin on TAM amount and repolarization was studied in animal models of lung cancer, renal cell carcinoma, breast cancer [220,221]. Endostatin improved tumor vessel normalization and maturation and promoted the repolarization of TAMs in mouse model of lung cancer [220]. When endostatin was administrated, the total amount of F4/80+ macrophages was significantly increased on day 10, the number of TAMs with pro-angiogenic properties (Tie-2-expressing) were reduced on day 6, and the amount of CD197+ M1-like TAMs increased after endostatin treatment [220]. In the model of BALB/c mice with renal cell carcinoma (RCC), circulating levels of M2-like markers and pro-angiogenic cytokines, including IL-4, IL-10, IL-13, and VEGF-A, were reduced in endostatin-treated animals in comparison with non-treated control [221]. In TAMs isolated from metastatic lungs of mouse treated with endostatin, the expression of M2 markers, IL-10, Arg-1, VEGF, and YM-1, was significantly reduced, while M1 markers IL-12 and iNOS did not change significantly in response to endostatin. Flow cytometry analysis revealed that endostatin treatment resulted in a decreased number of M2-polarized cells (positive for CD206, CD209, CD36 and arginase 1) and the reduction of the levels of IL-10-producing macrophages (F4/80+/CD206+/IL-10+, F4/80+/CD209+/IL-10+, and F4/80+/CD36+/IL-10+), but not the number of F4/80+ TAMs [221]. In tumors of BALB/c mouse breast cancer model, decrease in the average amount of TAMs and the number of M2-like F4/80+CD206+ TAMs and a significant increase in the number of M1-like F4/80+Nos2+ TAMs were observed after the treatment with endostatin [222]. The lumens of blood vessels were more regular and smoother, and the expression of VEGF and PIGF was decreased in the tumors of the endostatin-treated group [222]. Expression levels of the M1 markers Nos2, IL-12p40 and IL-6 were increased, and no changes in the expression of M2 markers (CD206, Arg1 and IL-10) was observed in mouse BMDMs and RAW264.7 cells transfected with the pEndostatin plasmid [222]. In a mouse experimental model of peritoneal sclerosis, the number of CD31+ blood vessels and F4/80+ macrophage accumulation were significantly inhibited by endostatin peptide [223]. 

In LLC xenograft mice, endostatin inhibited tumor angiogenesis by reducing the number of CD31+ cells and VEGF expression, aggravated hypoxia, and increased levels of inflammatory cytokines (IL-4, IL-6, IL-10) in tumor and CCL2 expression in endothelial cells and fibroblasts [224]. Contradictory to above listed examples, the percentage of F4/80+ macrophages was increased significantly after the treatment with recombinant human endostatin [224]. In patients with lung cancer treated with chemotherapy plus endostatin, a higher peripheral monocyte-to-lymphocyte ratio (MLR) was detected in patients who did not respond to the treatment (progressive disease) compared to the patients with partial regression [224].

Canstatin, a non-collagenous C-terminal fragment of type IV collagen α2 chain. Canstatin suppresses the proliferation, migration, and tube formation of vascular endothelial cells [225]. Western blot analysis demonstrated that canstatin inhibited the phosphorylation of Akt and induced the expression of FAS ligand expression in HUVEC cells [226]. In mouse model of renal cell carcinoma canstatin inhibited angiogenesis via decreasing of CD31+ cells [227]. However, we did not find any evidence of canstatin action on macrophages. 

Collected data demonstrate that, excluding its anti-angiogenic properties, endostatin can re-polarize TAMs to pro-inflammatory phenotype.

### 4.2. Indirect Inhibitors of Angiogenesis and TAMs

Indirect inhibitors of angiogenesis suppress the activity of pro-angiogenic factors produced by both cancer cells and cells of TME. We summarize the information concerning effective indirect inhibitors of angiogenesis in Table 3.

#### 4.2.1. Bevacizumab

The inhibition of VEGF signaling, a key mediator of angiogenesis in cancer, is the most common and effective anti-angiogenic strategy today. Bevacizumab is a recombinant humanized monoclonal antibody (mAb) that prevents the binding of circulating VEGF to its receptors [228].

Several studies have indicated that TAMs contributes to the resistance to the anti-angiogenic treatment based on VEGF targeting [5,229,230,231,232]. In patients with colorectal cancer treated with chemotherapy plus bevacizumab, low CD68+ TAM infiltration was predictive for high OS [5]. Bevacizumab increased the amount of non-classical M2b subpopulation CD11b+ CD86+ IL-10+ of TAMs in a triple-negative breast cancer (TNBC) model [229]. Patients with renal cell carcinoma treated with neoadjuvant bevacizumab demonstrated reduced CD31+ MVD and a reduced number of total CD68+, but not CD163+ TAMs in comparison with untreated patients [230].

In patients with recurrent glioblastoma (GBM) treated with anti-angiogenic therapy (AAT) (predominantly bevacizumab) plus chemotherapy, an increase in the numbers of CD11b+ myeloid cells, CD68+ total TAMs, and CD163+ M2-like TAMs was observed in autopsy specimens compared to initial diagnostic surgical specimens of the same patients [231]. The increase of the amount of the same cell populations was indicated in tumors of patients treated with AAT in comparison with patients treated only with chemotherapy [231]. In intracranial U87 xenografts, immunostaining revealed that bevacizumab-resistant glioblastoma showed an increased amount of TAMs and increased M2/M1 ratio, defined by M2 markers (Arg-1, TGF-β, MMP9) and M1 markers (NOS2, CXCL10, IL-1β), compared to bevacizumab-sensitive glioblastoma [232]. The possible mechanism of resistance to anti-angiogenic therapy can be bevacizumab-induced reduction of MIF expression in cancer cells resulting in the expansion of M2 macrophages, which in turn promotes tumor growth [232].

To overcome the resistance to bevacizumab, several approaches to combination therapy have been investigated. Inhibition of VEGF and ANG-2 with ANG-2/VEGF antibodies (CrossMab, A2V) diminished vessel density and tumor growth, and induced prolonged survival compared to anti-VEGF antibody (B20) alone in mice bearing orthotopic syngeneic (Gl261) GBM or human (MGG8) GBM xenografts [233]. A2V treatment induced reprogramming of M2 TAMs, defined as CD206high/CD11clow, toward the antitumor M1 phenotype (CD206low/CD11chigh) [233]. Dual inhibition of VEGFR/ANG-2 also demonstrated better effect than anti-VEGFR therapy alone (cediranib, a pan-VEGFR tyrosine kinase inhibitor). Such a therapeutic combination improved the normalization of vessels and reduced tumor burden [234]. Clodronate-containing liposomes (Clo-Lipo-DOTAP) depleted F4/80+ macrophages reduced MVD and the number of pulmonary nodules in B16/F10 lung metastatic melanoma model [235].

Several approaches for improvement of anti-angiogenic therapy include the additional inhibition of macrophage recruitment to the tumor. For example, combination therapy with bevacizumab and CCL2 inhibitor, mNOX-E36, decreased the recruitment of TAMs and angiogenesis, resulted in decreased tumor volume and blood volume in CCL2-expressing rat glioblastoma multiforme model [236]. Adding OLA-PEG, a novel CXCL-12 inhibitor, to bevacizumab or B-20 (anti-VEGF agent) significantly improved the anti-tumor effect by reducing intratumoral CD68+ TAM accumulation and by increasing the survival of tumor-bearing mice in the orthotopic G12 human glioblastoma model [237]. Altiratinib (a novel balanced inhibitor of MET/TIE2/VEGFR2) combined with bevacizumab reduced tumor volume, invasiveness, factor VIII-positive MVD, and Tie2+F4/80+ macrophage infiltration more effectively than bevacizumab alone [238]. The combination of agonistic anti-CD40 with antiangiogenic antibodies targeting two pro-angiogenic factors, VEGF-A and ANG2, facilitated tumor rejection and induced immune response in murine tumor models of colon cancer and melanoma [239]. Triple inhibition promoted pro-inflammatory macrophage skewing, significantly decreasing the proportion of CD206hiCD11clow M2-like TAMs and consequent increase of the M1/M2 ratio in these tumor models. Combined treatment also increased dendritic cell activation in the TME and promoted the intratumoral redistribution of cytotoxic CD8+ T cells in the tumors [239].

In summary, a number of mouse models and patients’ data indicate that anti-VEGF therapy results in the accumulation of TAMs in the tumor mass that can be a compensatory mechanism to supply the growing tumor with other pro-angiogenic factors produced by TAMs. Ant-angiogenic therapy combined with the inhibition of TAM recruitment or with TAM repolarization approaches can be more beneficial for cancer treatment. 

#### 4.2.2. Receptor Tyrosine Kinase (RTK) Inhibitors

Receptor tyrosine kinase (RTK) inhibitors include diverse drugs, such as axitinib, dasatinib, erlotinib, cetuximab, cediranib, imatinib, lenvatinib, regorafenib, sorafenib, and others. They are administered to patients with colorectal, lung, breast, renal, and other solid cancers characterized by specific histological and molecular-genetic subtypes. Below we present the information about the association of these drugs with TAM functions.

Axitinib, RTK inhibitor, significantly reduced tumor growth, followed by decreased number of TAMs (CD45+, CD11b+, F4/80+) in subcutaneous MC38 and LLC mouse models [240]. CD45+CD11b+Ly-6G−Ly6C+ monocytes were also significantly reduced by axitinib treatment in the spleen in MC38 and LLC tumors. The effect of axitinib was comparable to the effect of CCL2 neutralization together with VEGF-targeted therapy, suggesting that axitinib is effective as an inhibitor of myeloid cell differentiation [240].

Cetuximab, anti-EGFR monoclonal antibody, treatment reduced the number of CD206+ F4/80+ TAMs in AOM/DSS-induced colorectal cancer mouse model [241]. The expression of M2-like macrophage markers Arg1, MRC1 and IL-10, CCL17, and CCL22 were down-regulated, and the expression of M1-like macrophage markers iNOS, IL-12, and TNFa were induced by cetuximab in modeled TAMs, treated with CM of colon cancer cells in vitro [241,242]. In TAM-embedded breast cancer BT-20 spheroids with high EGFR density, treatment with cetuximab-targeted gold nanorods (CTX-AuNR) plus NIR irradiation enhanced ROS generation, induced cytotoxicity, and reprogrammed TAMs to the anti-tumor M1 phenotype [243].

Dasatinib, multiple kinase inhibitor, inhibited M2 polarization of TAMs in vitro [244,245]. Dasatinib improved cisplatin resistance in LLC cell lines A549R and H460R, decreased stemness and tumorigenesis of these cells by down-regulating Src, CD155 and MIF expression in vitro and in vivo [244]. Mannosylated mixed micelles delivered dasatinib (DAS-MMic) eliminated F4/80+ TAMs in 4T1 breast cancer model. DAS-MMic decreased CD31+ angiogenesis and the expression of major TAM-derived angiogenic cytokines, VEGF-A and MMP9 [246].

Erlotinib, EGFR inhibitor, in combination with bevacizumab/IFN (BVZ/IFN/ERLO) inhibited tumor growth, promoted blood vessel normalization and reduced lymphatic network in mouse RCC xenografts. This combination inhibited M2 polarization of macrophages by down-regulation of Arg1 and CD206 [247]. A novel erlotinib derivative, TD-92, reduced the number of pro-tumorigenic CD11b+ F4/80+ TAMs in LLC tumor model [248].

Imatinib significantly prevented M2-like polarization of BMDMs isolated from LLC mouse model, by inhibiting the expression of M2-like markers CD206, Arg1, Mgl2, MRC1, CDH1, and CCL2 in vitro. Imatinib reduced the amount of M2-polarized TAMs in tumor and decreased the number of metastases in an LLC subcutaneous model [249]. Administration of imatinib after agonistic anti-CD40 antibody activated TAMs, redirected TAMs to antitumor M1 phenotype, by increased TNF and IL-6 production through the NFκB pathway along with decreased IL-10 production in mouse model of gastrointestinal stromal tumor GIST [250]. Imatinib decreased the uptake of modified LDL and inhibited the activity of MMP-2 and MMP-9 in THP-1 macrophages [251]. 

Lenvatinib treatment decreased the number of CD31+ tumor blood vessels, diminished the amount of CD11b+F4/80+ TAMs and increased the percentage of activated CD8+ T cells secreting interferon (IFN)-γ+ and granzyme B (GzmB) in CT26 colon cancer mouse model. Lenvatinib plus anti-PD-1 combination treatment increased the number of CD8+ T cells and their cytotoxic activity in the CT26 model [252]. Lenvatinib in combination with golvatinib (E7050; c-Met, Tie2, and EphB4 inhibitor) reduced the CD31+ endothelial network, SMA+ pericyte network, and disrupted pericyte-mediated vessel stabilization, decreasing the interaction between ECs and pericytes in thyroid and endometrial cancer models [253]. Lenvatinib treatment increased the amount of F4/80+MRC1+ macrophages, while the lenvatinib/golvatinib combination negated that increase [253].

Regorafenib, multiple kinase inhibitor, decreased tumor CD31-positive angiogenesis, the total number of TAMs, but increased M1/M2 ratio and infiltration of tumor by CD4+ and CD8+ T cell in mouse model of HCC [254]. In BMDMs treated with regorafenib, the up-regulation of M1-like markers (TNFα, IL-6, MHC II) and down-regulation of M2-like markers (Arg1, CD206) were observed [254]. In mouse model of CRC regorafenib decreased tumor growth and tumor angiogenesis assessed as the number of Tie2-positive vessels, VEGFR2+/CD31+ area fraction and MVD [255]. Significantly decreased F4/80+ macrophage infiltration was found in regorafenib-treated CRC tumors [255].

Sorafenib is a small-molecule inhibitor of up to 40 kinases, potently inhibiting pro-angiogenic receptor tyrosine kinases including VEGFR-1/2/3, PDGFR-β, and FGFR1 [256]. Immunological effects of sorafenib treatment were demonstrated for hepatocellular carcinoma (HCC) in several studies. Thus, in patients with HCC the number of CD68+ TAMs and EMT-related proteins (fibronectin and vimentin) was reduced after sorafenib treatment [257]. In vitro sorafenib decreased the expression of EMT-related genes (Vimentin, Snail, and Slug) and migration of HepG2 cells stimulated with CM of activated THP-1 macrophages via blocking HGF-Met signaling in HepG2 cells [257]. Oppositely, when THP-1 macrophages were polarized to M2, they accumulated in sorafenib-treated HCC xenograft model in vivo, and promoted proliferation, colony formation and migration of HCC cells in vitro by producing abundant HGF [256]. Sorafenib induced the pro-inflammatory activity in TAMs isolated from HCC tissue of transgenic mice by enhancing IL-12 secretion or pyroptosis in macrophages [258,259]. Sorafenib-activated pro-inflammatory TAMs triggered antitumor NK cell response against HCC target cells by increased degranulation and IFN-γ secretion [259]. 

Several strategies for combination of TAM-targeting and sorafenib treatment were investigated. Novel Chinese medicine formula, compound Kushen injection (CKI), in combination with sorafenib activated pro-inflammatory response in TAMs and diminished immunosuppression in HCC tumors [260]. In HCC mouse model, CKI plus sorafenib increased M1(iNOS)/M2(Arg1) ratio and decreased M2 distribution leading to the activation of cytotoxic ability of CD8+ T cells [260]. When concomitant treatment with liposomal clodronate was applied against TAMs, no obvious anti-tumor effect of sorafenib+CKI was detected anymore in comparison with liposomal clodronate, indicating that mechanism of action of sorafenib is more likely associated with immunomodulatory activity of TAMs [260]. TAM-targeting probe for diagnostic imaging and treatment of tumors was synthesized by conjugating a monoclonal anti-CD206 antibody with a near-infrared phthalocyanine dye (IRD-αCD206). IRD-αCD206 allowed to visualize the recruitment of M2 macrophages in tumor after sorafenib treatment and, upon light irradiation, suppressed tumor growth and inhibited lung metastasis in a mouse model of 4T1 tumor [261]. It was demonstrated that monocyte/macrophages are enriched in the perivascular areas of CXCR4+ vessels in HCC [262]. In vitro CM from tumor-exposed human monocytes promoted TNF-α-induced CXCR4 expression on HUVECs via the Raf-Erk pathway. Simultaneous depletion of TAMs with zoledronic acid and treatment with sorafenib inhibited primary tumor growth and lung metastasis in an orthotopic HCC model through the reduction of the CXCR4+ vascular density [262]. Thus, the possible anti-tumor mechanism of sorafenib action is directed to the re-orientation of immune cells in tumor followed by pro-inflammatory TAM activation.

Likewise, RTK inhibitors demonstrated significantly reduced tumor growth and activated blood vessel normalization in numerous cancer models in vivo. This effect is achieved by the modulation of the immune system and induction of protective anti-tumor immunity that is mediated by TAM depletion and re-polarization in tumor. 

#### 4.2.3. Celecoxib (Anti-COX2)

Celecoxib is a selective COX-2 inhibitor that is widely used in arthritis treatment [263]. Besides, celecoxib has antitumor activity and suppresses the proliferation, migration, and invasion of tumor cells in different cancers, including bladder cancer [264], pancreatic cancer [265], breast cancer [266], oral squamous cell carcinoma [267], and colorectal adenomas [268]. Antitumor effect of celecoxib can be attributed to the suppression of tumor angiogenesis through inhibition of COX-2-related signaling pathways. For example, in mouse H22 hepatocarcinoma model, inhibition of COX-2 by celecoxib reduced tumor growth and MVD through inhibition of PTEN/PI3K/Akt/HIF-1 signaling pathway in tumor cells [269]. Celecoxib suppressed tumor growth and angiogenesis, which mediated a decrease in the amount of CD34+ cells, inhibition of COX-2, PGE2 synthesis, and VEGF and MMP-2 mRNA expression in a mouse model of colorectal cancer [270]. 

The anti-angiogenic activity of celecoxib can be associated with macrophage reprogramming from M2 to M1 phenotype. Celecoxib suppressed the migration and invasiveness of gastric cancer cells stimulated by M2-polarized THP-1 macrophages in vitro [271]. In ApcMin/+ mouse polyps, celecoxib up-regulated mRNA levels of M1-related genes (iNOS and CXCL10) and down-regulated levels of M2-related genes (Arg1, Ym1, MR, and Trem2) resulting in the changing of TAM phenotype from M2 to M1 [272]. Celecoxib also reduced size and number of polyps in IFN-γ-dependent mechanism [272]. Celecoxib in combination with IFNγ reduced the expression of MMP-2, MMP-9, and VEGF-A in tumor and decreased MVD, mediated by the increased amount of CD68+iNOS+ M1 macrophages and by decreased amount of CD68+Arg1+ M2 macrophages in mouse model of LLC [273]. 

Considering COX-2 as one of the crucial regulators of tumor angiogenesis and TAMs as a main source of COX-2 in the TME, we suggested that the administration of celecoxib in combination with RTK inhibitors can be efficient therapeutic strategy for cancer treatment.

**Table 3:** The effect of FDA-approved anti-angiogenic therapy on macrophage activity.

Therapeutic Drug/Combination	Targets	Macrophage Activity	Experimental Model
Bevacizumab	mAb against VEGF	Increases amount of M2-like TAMs after treatment	Breast cancer and glioblastoma model, patients with glioblastoma [229,231,232]
CrossMab, A2V	mAb against Ang-2/VEGF	Diminishes MVD and tumor growth, and induces prolonged survival, induces re-programming of pro-tumor M2 TAMs to M1-like TAMs	Mice bearing orthotopic syngeneic (Gl261) or human (MGG8) gioblastoma xenografts [233]
Bevacizumab plus CCL2 inhibitor (mNOX-E36)	mAb against VEGF+CCL2 inhibitor	Decreases the recruitment of TAMs, tumor volume and blood volume	CCL2-expressing rat glioblastoma multiforme model [236]
Bevacizumab plus OLA-PEG	mAb against VEGF+CXCL-12 inhibitor	Reduces accumulation of CD68+ TAMs and increases the survival of tumor-bearing mice	Orthotopic G12 human glioblastoma model [237]
Bevacizumab plus Altiratinib	mAb against VEGF+inhibitor of MET/TIE2/VEGFR2	Reduces tumor volume, invasiveness, MVD, and Tie2+/F4/80+ macrophage infiltration	Glioblastoma mouse model [238]
Triple inhibition (anti-CD40, anti-VEGF-A and anti-Ang2)	anti-CD40, anti-VEGF-A and anti-Ang2	Promotes pro-inflammatory macrophage skewing, decreasing the proportion of CD206^hi^CD11c^low^ M2-like TAMs and increasing the M1/M2 ratio, and facilitates tumor rejection	Murine tumor models of colon cancer and melanoma [239]
Axitinib	TKR inhibitor	Reduces tumor growth, decreases number of TAMs	Subcutaneous MC38 and LLC mouse models [240]
Cediranib plus MEDI3617 (an anti-Ang-2–neutralizing antibody)	VEGFR inhibitor+anti-Ang2	Reduces tumor growth, induces morphological normalization and TAM-mediated improved survival	Murine glioblastoma models [234]
Cetuximab	mAb against EGFR	Reduces the number of CD206+F4/80+ TAMs, increases expression of M1-like markers and decreases expression of M2-like markers	AOM/DSS-induced colorectal cancer mouse model [241], modeled TAMs, treated with conditioned medium of colon cancer cells in vitro [241,242]
Cetuximab-targeted gold nanorods (CTX-AuNR) plus NIR irradiation	mAb against EGFR	Enhances ROS generation, and re-programms TAMs to the anti-tumor M1 phenotype	TAM-embedded breast cancer BT-20 spheroids [243]
Celecoxib	COX-2 inhibitor	Changes TAM phenotype from M2 to M1	Apc^Min/+^ mouse polyps [272]
Celecoxib plus IFNγ	COX-2 inhibitor	Decreases MVD, increases amount of CD68+iNOS+ M1 macrophages and decreases amount of CD68+ARG1+ M2 macrophages	Mouse model of LLC [273]
Dasatinib	TKR inhibitor	Inhibites M2 polarization of TAMs	In vitro [244,245]
Mannosylated mixed micelles delivered dasatinib (DAS-MMic)	TKR inhibitor	Eliminates F4/80+ TAMs, decreases CD31+ angiogenesis	4T1 breast cancer model [246]
Etoricoxib	COX-2 inhibitor	Suppresses MVD and the infiltration of macrophages	Mouse model of melanoma [59]
Erlotinib plus bevacizumab/IFN (BVZ/IFN/ERLO)	EGFR inhibitor+anti-VEGF	Inhibites tumor growth, promotes blood vessel normalization, reduces lymphatic network, and inhibites M2 polarization	Mouse xenografts of RCC [247]
Erlotinib derivative, TD-92	EGFR inhibitor	Reduces the number of pro-tumorigenic CD11b+F4/80+ TAMs	LLC tumor model [248]
Imatinib	TKR inhibitor	Prevents M2-like polarization of BMDMs, reduces amount of M2-polarized TAMs	LLC mouse model [249]
Imatinib plus anti-CD40 antibody	TKR inhibitor+anti-CD40	Redirects TAMs to antitumor M1 phenotype	Mouse model of GIST [250]
Lenvatinib	RTK inhibitor	Decreases the number of CD31+ tumor blood vessels, diminishes the amount of CD11b+F4/80+ TAMs and increases the percentage of activated CD8+ T cells	CT26 colon cancer mouse model [252]
Lenvatinib plus golvatinib	RTK inhibitor+c-Met, Tie2, and EphB4 inhibitor	Disrupts pericyte-mediated vessel stabilization, reduces angiogenesis, decreases the amount of F4/80+MRC1+ macrophages	Thyroid and endometrial cancer models [253]
Regorafenib	TKR inhibitor	Decreases tumor angiogenesis, the total number of TAMs, and increases M1/M2 ratio and infiltration of tumor by CD4+ and CD8+ T cell	Mouse model of HCC [254], mouse model of CRC [255]
Sorafenib	small molecule, RTK inhibitor	Reduces the number of CD68+ TAMs, induces pro-inflammatory activity in TAMs	Patients with HCC [257], mouse model of HCC [258,259]
Sorafenib plus compound Kushen injection (CKI)	RTK inhibitor+natural compound	Increases M1/M2 ratio, decreased M2, activates cytotoxic ability of CD8^+^ T cells	HCC mouse model [260]
Sorafenib plus IRD-αCD206	RTK inhibitor+anti-CD206	Suppresses tumor growth and inhibites lung metastasis	Mouse model of 4T1 tumor [261]
Sorafenib plus zoledronic acid	RTK inhibitor+TAM-depleting agent	Suppresses tumor growth and inhibites lung metastasis	Orthotopic HCC model [262]

EC—endothelial cell; GIST—gastrointestinal stromal tumor; HCC—hepatocellular carcinoma; LLC—Lewis lung carcinoma; mAb—monoclonal antibody; MVD—microvessel density; RCC—renal cell carcinoma; RTK—receptor tyrosine kinase; TAM—tumor-associated macrophage; TEM—Tie2-expressing monocyte/macrophage.

## 5. Conclusions

The limited efficacy of anti-angiogenic therapy can be explained by the existence of multiple regulators of angiogenesis that are not taken into consideration by the current therapeutic approaches. A number of positive and negative regulators of angiogenesis in the tumor microenvironment are produced by TAMs. Accumulating evidence indicates that, apart from well-known angiogenic factors (VEGF-A, PDGF, Ang-1 and -2, MMPs, PA), there are plenty of novel angiogenesis-regulating proteins that belong to different classes. In the context of TAMs, essential factors that control tumor angiogenesis include members of the SEMA family, S100A family, chitinase-like proteins, osteopontin, SPARC, COX-2, Tie2, and others. Despite the fact that some of these soluble mediators can be produced by cancer cells, TAMs are a major source producing a broad spectrum and high amount of pro-angiogenic regulators, while some factors can be also unique for TAMs. Extremely limited is our knowledge about the genetic variants of novel classes of angiogenesis regulators that can affect tumor vascularization and sensitivity to therapy. Most of the knowledge we have concerns the genetics of VEGF. However, even this is not carefully investigated in the context of macrophages, which are major VEGF producers in cancer. Considering that the functional status of macrophages is tightly regulated by the epigenetic mechanisms, the epigenetics of pro-angiogenic factors also have to be understood to efficiently block the pro-angiogenic program of TAMs by therapeutic tools.

Numerous in vivo studies revealed that combination of TAM-targeted agents with anti-angiogenic drugs improve the efficacy of the treatment. Thus, in order to increase the efficacy of anti-angiogenic therapy, the development of complementary approaches that combine the agents that target alternative mechanisms of blood vessel formation and re-program TAMs is needed.

Targeting of YKL-40, SEMA3a, and S100A4 was already assessed in the animal models. Anti-YKL-40 monoclonal antibody inhibited angiogenesis and tumor progression in a murine glioblastoma model [274]. Recently, anti-human YKL-40 mAb were developed that inhibit tumor growth in a murine B16F10 melanoma model [275]. However, in a human melanoma xenograft model, anti-YKL-40 mAb was not successful and resulted in increased tumor growth [276]. In a glioblastoma model, treatment with anti-SEMA3A F11 antibody exhibited a notable tumor inhibitory effect and TAM infiltration in vivo [277]. Neutralizing monoclonal antibody 5C3 against S100A4 decreased endothelial cell migration, tumor growth and angiogenesis in immunodeficient mouse xenograft models of pancreatic cancer and melanoma [30]. Anti-S100A4 mAb treatment significantly reduced metastatic burden in the lungs of a mammary carcinoma model by blocking the recruitment of T cells to the site of the primary tumor [278]. Clinical trials were performed concerning anti-COX2 agent celecoxib combined with standard therapy for the treatment of colorectal, breast, lung, prostate, gastric, and head and neck cancers [266].

The application of genetically engineered macrophages (GEM) is a promising immunotherapeutic strategy in cancer treatment [279,280]. GEM are modified by recombinant viral-based technology. The main advantages of GEM application are the suppression of tumor development through supporting T-cell response and formation of pro-inflammatory TME increasing tumor cell death [279,280,281]. The success made with this technology is promising for the suppression of the pro-angiogenic potential of TAMs. However, genetic engineering has to target a complex system of several pro-angiogenic factors to overcome the compensation mechanisms.

Tools for the targeting of other factors still have to be developed. Moreover, a novel anti-angiogenic therapy has to be elaborated in the context of chemotherapy and rapidly developing immunotherapy, since TAMs cooperate with these approaches and define their efficiency. A combined therapy that considers multiple activities of TAM is the strategy to personalize cancer treatment and to achieve maximum efficiency with minimal relapse risk. 

## Data Availability

All data are available online with common access. The data analyzed during the current study are available from the corresponding author on reasonable request.

## Abbreviations

TAMs	Tumor-associated macrophages
BMDM	Bone marrow-derived macrophage
DC	Dendritic cell
PFS	Progression-free survival
LLC	Lewis lung carcinoma
RCC	Renal cell carcinoma
OSCC	Oral squamous cell carcinoma
mAb	monoclonal antibody
MVD	Microvessel density
CM	Conditioned medium
EC	Endothelial cell
CAM	Chick chorioallantoic membrane
CLP	Chitinase-like protein
OPN	Osteopontin (SPP1)
OSN	Osteonectin, Secreted protein acidic and rich in cysteine (SPARC)
NSCLC	Non-small cell lung cancer
TME	Tumor microenvironment
SMA	Smooth muscle actin
HUVEC	Human umbilical vein endothelial cell
PDAC	Pancreatic ductal adenocarcinoma cell
WT	Wild type
